# Evolutionary remodeling of the ventral retina enables aerial vision in the four-eyed fish Anableps anableps

**DOI:** 10.1038/s41467-026-78775-4

**Published:** 2026-10-14

**Authors:** Louise Perez, Lina Shi, Alyssa Daspit, Keyla N. Pruett, Carsyn Smith, Anna Prideaux, Josane F. Sousa, Patricia N. Schneider

**Affiliations:** Department of Biological Sciences, Louisiana State University, Baton Rouge, LA US

**Keywords:** Evolutionary developmental biology, Non-model organisms, Developmental neurogenesis, Retina

## Abstract

The emergence of evolutionary novelties remains a central challenge in biology, particularly for complex organs such as the vertebrate eye. Here, we use the four-eyed fish *Anableps anableps* to investigate adaptation to simultaneous aerial and aquatic vision. Single-nucleus and spatial transcriptomics reveal that the ventral retina, which processes aerial light, is developmentally expanded and exhibits region-specific opsin expression. Ventral PR1 cones express *opn1mw2* rather than the longer-wavelength-sensitive *opn1lw* paralogs expressed dorsally. Machine-learning predictions indicate that Opn1mw2 is red-shifted relative to Opn1mw1 but remains blue-shifted relative to dorsal Opn1lw pigments. Dorsal-ventral patterning markers further reveal an overrepresentation of ventral retinal identities. Comparative spatial profiling of developing and adult *Anableps* and killifish indicates dorsal displacement of the retinal boundary, producing a proportionally larger ventral domain. Thus, *Anableps* evolved aerial vision through developmental expansion of the ventral retina and regional reconfiguration of opsin expression within conserved cone subtypes.

## Abstract (web summary)

Using single-nucleus RNA-sequencing and spatial transcriptomics, researchers showed the four-eyed fish Anableps evolved aerial vision through developmental expansion of its ventral retina and regional opsin gene tuning.

## Introduction

**Fig. 1:**
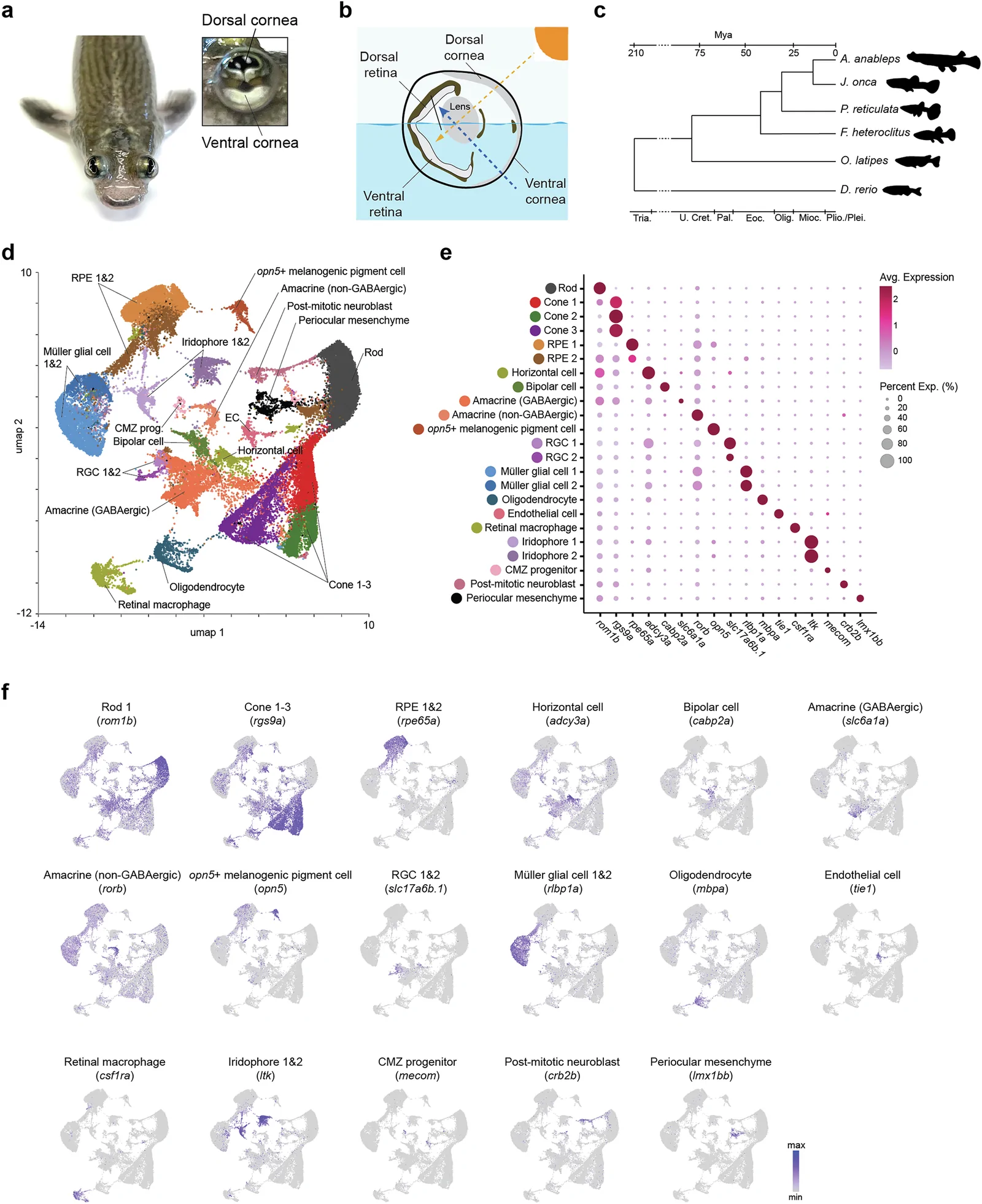
SnRNA-seq reveals cellular landscape of adult *Anableps* retina. **a** Dorsal view of adult *Anableps* head, with an inset showing a lateral view of the eye, highlighting the dorsal and ventral corneas. **b** Schematic representation of the visual field: aerial input (yellow arrow) through the dorsal cornea and aquatic input (blue arrow) through the ventral cornea. **c** Phylogenetic relationship of *Anableps* relative to representative species of Anablepidae and other Cyprinodontiformes. **d** UMAP plot shows automatic clustering of snRNA-seq gene expression profiles, revealing 23 distinct cell clusters. **e** Dot plot showing expression of gene markers of retinal cell types identified in our dataset. **f** UMAP plots show expression of representative gene markers for select retinal cell types.

While many vertebrate eye specializations involve reduction or loss of visual structures, as seen in cavefishes^1^, species within the *Anableps* genus present a striking example of anatomical and functional innovation. The four-eyed fish swims at the waterline and, despite its common name, possesses only two eyes, each divided into dorsal and ventral optical regions specialized for aquatic and aerial vision, respectively. This unique ocular morphology includes split pupils and corneas (Fig. 1a), a pyriform lens, and a single retina that simultaneously receives visual input from both aerial and aquatic environments^2–4^ (Fig. 1b). Phylogenetically, *Anableps* belongs to a clade of Cyprinodontiform fishes, including the one-sided livebearer (*Jenynsia onca*), the killifish (*Fundulus heteroclitus*), the guppy (*Poecilia reticulata*) and the medaka (*Oryzias latipes*), all of which are fully aquatic and possess typical teleost retinas (Fig. 1c). Therefore, the unique ocular morphology of *Anableps* evolved within a lineage otherwise characterized by conventional aquatic vision, sometime the split between *Anableps* and its sister lineage *Jenynsia* to the Miocene, ~12.5 Mya^5–7^ (Fig. 1c). In most teleosts, the retina is regionally specialized along the dorsoventral axis to match the optical properties of the aquatic environment. In species such as the zebrafish (*Danio rerio*), the ventral retina receives downwelling light filtered through the water column, which is enriched in longer wavelengths, and accordingly expresses red-shifted opsin paralogs such as *opn1mw4* and *opn1lw1*^8^. This configuration optimizes color sensitivity for the underwater visual field. In *Anableps*, however, the ventral retina faces the aerial environment, while the dorsal retina continues to process aquatic light.

A previous survey of photoreceptor absorbance spectra in *Anableps* reported no difference in visual pigment composition between the dorsal and ventral retina^9^. However, subsequent in situ hybridization analyses suggested a more complex pattern, with ultraviolet (UV)- and short-wavelength-sensitive opsins distributed across the retina, middle-wavelength-sensitive opsin expression confined to the ventral region, and long-wavelength-sensitive opsin expression restricted to the dorsal region^3^. These seemingly conflicting findings have left the spatial organization and functional specialization of cone types in *Anableps* unresolved.

Anatomical studies have revealed that the *Anableps* ventral retina contains a denser ganglion cell population, consistent with enhanced sampling of the aerial visual field^10^. Similarly, histological analyses indicate that the ventral retina is thicker than the dorsal retina, suggesting a higher overall cell density in this region^4^. Furthermore, the middle wavelength opsin encoded by *opn1mw1* gene was found to be expressed in the entire ventral half of the retina^3^. Therefore, a central question is whether the putatively enlarged ventral half of the *Anableps* retina arose through an expansion of ventrally specified cell types, or through functional reprogramming of developmentally dorsal retinal domains. In the first scenario, ventral cell identities defined by canonical ventral patterning markers would have expanded to occupy a disproportionate portion of the retina. Alternatively, the dorsal retina, while retaining its molecular dorsal identity, may have acquired opsin expression profiles and photoreceptor specializations suited for aerial light detection. Distinguishing between these possibilities is essential to understanding how developmental patterning and photoreceptor diversification contributed to this evolutionary novelty.

Here, we use a multi-omics approach to investigate how the *Anableps* retina was remodeled to detect and process simultaneous aerial and aquatic light stimuli. Using single-nucleus RNA sequencing (snRNA-seq), we identified the major retinal cell types comprising the *Anableps* retina and their molecular markers. Spatial transcriptomic profiling (spatial RNA-seq) of the *Anableps* eye allowed us to map gene expression profiles to their respective anatomical locations. Comparative gene synteny analyses confirmed that the *Anableps* opsin gene repertoire was already present in the common ancestor shared with guppies. Interestingly, whereas zebrafish exhibits marked dorsal-ventral differences in cone subtype distribution and opsin expression, the *Anableps* ventral retina contains all four ancestral cone subtypes, PR1-PR4, with region-specific opsin expression. Furthermore, expression analysis of dorsal-ventral patterning markers showed that ventral-type cells predominate in the *Anableps* retina, suggesting an expansion of cells specialized for aerial light processing. Assessment of opsin spectral tuning using OPTICS revealed that *Anableps* Opn1mw2 is red-shifted relative to other middle-wavelength-sensitive pigments, potentially extending the sensitivity of ventral PR1 cones toward longer wavelengths without reaching the predicted *λ*_max_ of dorsal Opn1lw pigments. In addition, we show via spatial RNA-seq how the dorsal and ventral retinal domains are established during eye development and maintained in adulthood. Finally, comparison of *Anableps* and killifish spatial RNA-seq datasets indicated a dorsal shift in the retina dorsal-ventral boundary in *Anableps*, effectively enlarging the ventral retinal territory. Together, these findings demonstrate that *Anableps* evolved an expanded ventral retinal domain with regionally reconfigured opsin expression, providing a developmental and molecular basis for aerial vision.

## Results

### snRNA sequencing identifies the retinal cell populations underlying dorsal-ventral specialization

To determine how the dorsal and ventral retina differ in cellular composition and photoreceptor identity, we performed single-nucleus RNA sequencing of adult *Anableps* retinas using a combinatorial barcoding approach (Parse Biosciences, 2024). Retinal nuclei were isolated from five independent biological replicates of whole adult retinas. Trailmaker (Parse Biosciences, 2024) and RStudio were used to assess the quality control metrics, preprocess the data, and complete our snRNA-seq analysis. After preprocessing, we retained 47,586 high-quality nuclei for downstream analyses (Supplementary Fig. 1). Unbiased clustering identified 23 transcriptionally distinct cell populations that were visualized by uniform manifold approximation and projection (UMAP) and annotated using canonical retinal marker genes, revealing photoreceptors, interneurons, and non-neuronal supporting cells (Fig. 1d). Among photoreceptor cells, the initial whole-retina analysis identified one rod subtype (*rom1b*^+^) and three cone subtypes (*rgs9a*^+^) (Fig. 1d, e). The retinal interneuron cells included a horizontal cell cluster (*adcy3a*^+^), a bipolar cell cluster (*cabp2a*^+^), and two amacrine cell clusters, GABAergic (*slc6a1a*^+^) and non-GABAergic (*rorb*^+^). The other clusters were characterized by two subtypes of retinal pigment epithelium (RPE) (*rpe65a*^+^), one *opn5*+ melanogenic pigment cell (*opn5*^+^), two subtypes of retinal ganglion cells (RGCs) (*slc17a6b.1*^+^), two subtypes of Müller glia cells (*rlbp1a*^+^), one subtype of oligodendrocyte (*mbpa*^+^), endothelial cells (*tie1*^+^), retinal macrophage (*csf1ra*^+^), two subtypes of iridophores (*ltk*^+^), CMZ progenitor (*mecom*^+^), post-mitotic neuroblast (*crb2b*^+^), and periocular mesenchyme (*lmx1bb*^+^) (Fig. 1d, e). UMAP plots showed gene marker expression within expected cell populations, including rod (*rom1b*), cones (*rgs9a*), horizontal cells (*adcy3a*), bipolar cells (*cabp2a*), amacrine cells: GABAergic (*slc6a1a*) and non-GABAergic (*rorb*), *opn5*+ melanogenic pigment cell (*opn5*), RGC (*slc17a6b.1*), Müller glia cell (*rlbp1a*) oligodendrocyte (*mbpa*), endothelial cell (*tie1*), retinal macrophage (*csf1ra*), iridophore (*ltk*), CMZ progenitor (*mecom*), post-mitotic neuroblast (*crb2b*), and periocular mesenchyme (*lmx1bb*) (Fig. 1f). These data established the cellular framework for subsequent analyses of dorsal-ventral patterning, cone subtype distribution, and region-specific opsin expression underlying aerial vision in *Anableps*.

### Spatial transcriptomics localizes retinal cell populations within the dorsal and ventral retina

**Fig. 2:**
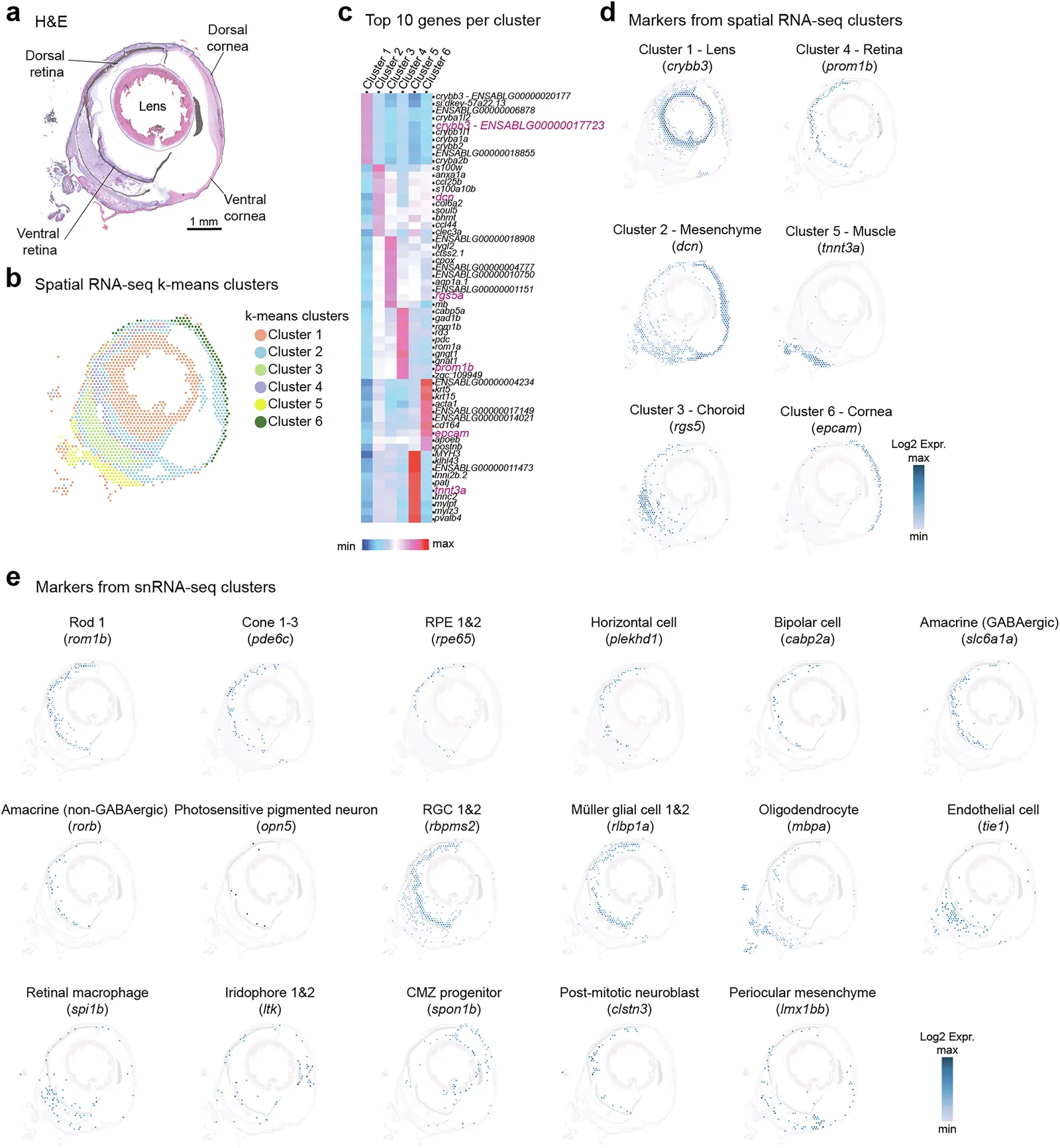
Spatial transcriptomic profiling of the adult *Anableps* eye. **a** Histological section used for spatial RNA-seq of the *Anableps* eye. **b** Unbiased *k*-means clusters identify 6 different gene-expression territories in the *Anableps* eye. **c** Heatmap showing the top 10 differentially expressed genes per cluster. **d** Eye gene markers from spatial RNA-seq clusters: *crybb3* (lens), *dcn* (mesenchyme), *rgs5* (choroid), p*rom1b* (retina), *tnnt3a* (muscle), and *epcam* (cornea). **e** Retina gene markers from our snRNA-seq clusters: *rom1b* (rod), *pde6c* (cone 1-3), *rpe65* (Retinal pigment epithelium (RPE) 1&2), *plekhd1* (horizontal cell), *cabp2a* (bipolar cell), *slc6a1a* (amacrine GABAergic), *rorb* (amacrine non-GABAergic), *opn5* (*opn5*+ melanogenic pigment cell), *rbpms2* (Retinal ganglion cells-RGC 1&2), *rlbp1a* (Müller glial cells 1&2), *mbpa* (oligodendrocyte), *tie1* (endothelial cell), *spi1b* (retinal macrophage), *ltk* (iridophore cells 1&2), *spon1b* (Ciliary marginal zone (CMZ) progenitor), *clstn3* (post-mitotic neuroblast), and *lmx1bb* (periocular mesenchyme). Scale bar = 1 mm.

Our snRNA-seq analysis revealed 23 transcriptionally distinct retinal cell clusters. To determine how these molecularly defined cell types are spatially arranged within the *Anableps* retina, we conducted spatial RNA-seq of the whole adult *Anableps* eye (Fig. 2a, b) using the Visium platform (10x Genomics). Unsupervised k-means clustering (*k* = 6) using Loupe Browser v8.1.2 (10x Genomics) identified transcriptionally distinct clusters, and assessment of the top ten genes differentially expressed in each cluster identified characteristic marker genes (Fig. 2c and Supplementary data 1). Based on these markers, we assigned clusters to their likely tissue identities: lens (*crybb3*), mesenchyme (*dcn*), choroid (*rgs5*), retina (*prom1b*), muscle (*tnnt3a*), and cornea (*epcam*) (Fig. 2b–d). This spatial RNA-seq atlas established a framework for mapping additional cell-type-specific markers across the *Anableps* eye.

To validate that the retinal cell-type markers identified in our snRNA-seq analysis corresponded to their expected anatomical locations, we examined their spatial distribution within the *Anableps* eye. Although 10x Genomics Visium v1 slide architecture does not achieve single-cell resolution, as each capture spot encompasses transcripts from multiple neighboring cells (approximately 5–10 cells of the *Anableps*), the dataset nonetheless enabled robust localization of major retinal cell types across tissue layers. Expression of *rom1b* (rod), *pde6c* (cones 1–3), *plekhd1* (horizontal cell), *cabp2a* (bipolar cell), *slc6a1a* (amacrine-GABAergic), *rorb* (amacrine non-GABAergic), *opn5* (*opn5*+ melanogenic pigment cell), *rbpms2* (RGC), *rlbp1a* (Müller glia cell) and *clstn3* (post-mitotic neuroblast) was confined to the retinal region, consistent with their known laminar organization. In contrast, *tie1* (endothelial cell), *spi1b* (retinal macrophage), *ltk* (iridophore), and *lmx1bb* (periocular mesenchyme) were enriched in the choroid. Expression of *mbpa* (oligodendrocyte) localized to the muscle underlying the sclera and adjacent mesenchyme, and *spon1b* (CMZ progenitor) was expressed in the CMZ, cornea, and around the lens (Fig. 2e). Together, these results establish the spatial framework for subsequent analyses of dorsal-ventral retinal organization, cone subtype distribution, and region-specific opsin expression in *Anableps*.

### Conserved cone subtypes exhibit region-specific opsin expression in *Anableps*

**Fig. 3:**
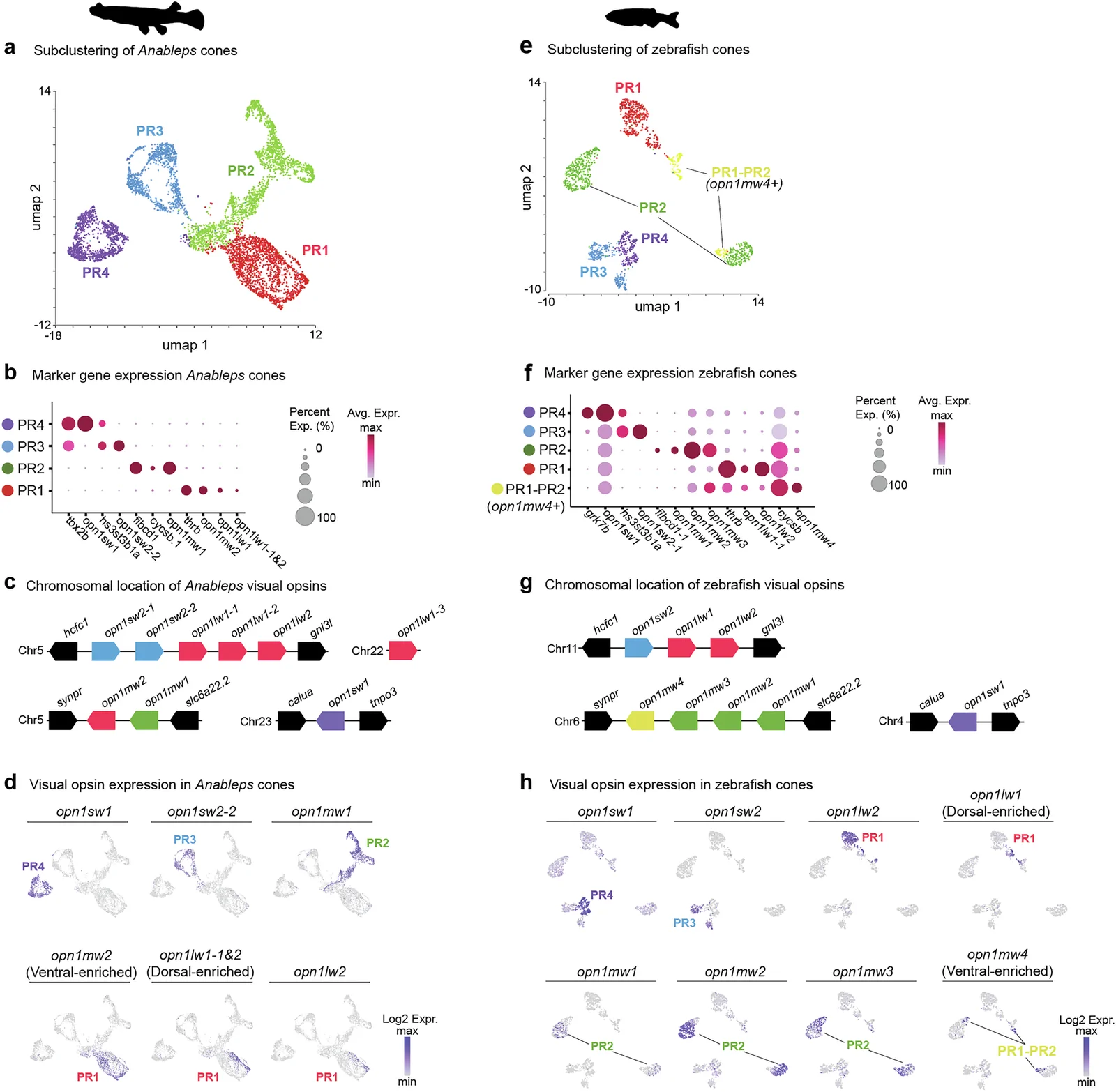
Comparison of cone cell diversity and opsin gene expression in *Anableps* and zebrafish retinas. **a**,**e** UMAP plots showing cone subclusters in *Anableps* (**a**), and zebrafish (**e**). **b**,**f** Dot plots of cone subtype gene markers and visual opsin genes in *Anableps* (**b**) and zebrafish (**f**). **c**,**g** Chromosomal organization of *Anableps* (**c**) and zebrafish (**g**) visual opsin genes. **d**,**h** UMAP plots showing the expression patterns of visual opsins in *Anableps* (**d**) and zebrafish (**h**) cones.

To compare cone photoreceptor diversity and opsin gene expression between *Anableps* and zebrafish, we subclustered cone cells from our *Anableps* snRNA-seq dataset and a publicly available single-cell RNA-seq (scRNA-seq) dataset from adult zebrafish retina^8^. Subclustering of cone nuclei resolved four cone subtypes in *Anableps* (Fig. 3a, b and Supplementary Fig. 2a) and five cone subtypes in zebrafish (Fig. 3e, f and Supplementary Fig. 2b). In *Anableps*, cone subtypes were identified using subtype-associated markers^3,8^: PR4, marked by *tbx2b* and expressing *opn1sw1* (SWS1); PR3, expressing *hs3st3b1a* and *opn1sw2-2* (SWS2b); PR2, expressing *fibcd1a* and *opn1mw1* (RH2-2); and PR1, marked by *thrb* and expressing either *opn1mw2* (RH2-1) or the *opn1lw* paralogs (LWS1&2) (Fig. 3a, b). Similarly, zebrafish cones comprised PR4 (*grk7b*^*+*^, *opn1sw1*^+^; SWS1), PR3 (*hs3st3b1a*^*+*^*, opn1sw2*^+^; SWS2), PR2 (*fibcd1-1*^*+*^*, opn1mw1*^+^; RH2-1/*opn1mw2*^+^; RH2-2/*opn1mw3*^+^; RH2-3), and PR1 (*thrb*^*+*^, *opn1lw1*^+^; LWS1/*opn1lw2*^+^; LWS2) cones (Fig. 3a, b). Consistent with previous reports, zebrafish *opn1mw4* (RH2-4) was expressed in a unique population of double cones previously classified as ventral retina PR1-PR2 cones expressing high levels of *cycsb*^8^ (Fig. 3e, f). Likewise, in agreement with previous findings^3^, we did not detect significant expression of *Anableps* opn1sw2-1 (SWS2a) or *opn1lw1-3* (Fig. 3b–d; Supplementary Fig. 2).

The availability of a high-quality *Anableps* genome assembly (fAnaAna1) allowed us to re-examine the opsin gene repertoire and chromosomal organization. We confirmed nine visual opsin genes^3,11^ in the *Anableps* genome (Fig. 3c), contrasting to the eight visual opsins of zebrafish (Fig. 3g)^12^. Although the number of opsin paralogs in *Anableps* differs from zebrafish, their counterparts are present in the guppy and the one-sided livebearer^3,13^. Conserved synteny analyses confirmed that the *Anableps* opsin gene complement was already present in the common ancestor shared with killifish (Supplementary Fig. 3a–c).

Finally, mapping visual opsin expression across cone subclusters revealed a clear correspondence between opsin identity and cone subtype in both species (Fig. 3d, h). In both zebrafish and *Anableps*, several cone subtypes showed additional transcriptional subdivision, although this pattern was more pronounced in *Anableps* (Fig. 3d, h). Notably, in *Anableps*, the ventral-enriched *opn1mw2* and dorsal-enriched *opn1lw1-1&2* expression patterns^3^ distinguished two regional PR1 cone populations (Fig. 3d). Together, these findings are consistent with the interpretation that the transcriptional subdivision of PR1 cones reflects ventrally and dorsally localized photoreceptor populations with distinct opsin expression patterns.

### Expanded representation of cone subtypes in the *Anableps* ventral retina

To assess how cone subtypes are distributed along the dorsal-ventral axis of the *Anableps* and zebrafish retinas, we classified cone cells according to established dorsal-ventral markers. Our *Anableps* snRNA-seq dataset captures nascent nuclear transcripts, which are generally less abundant than cytoplasmic mRNAs measured in scRNA-seq. This limits the detection of lowly expressed transcription factors such as the dorsal marker *tbx5b* and ventral marker *vax2*^14^. We confirmed by hybridization chain reaction RNA fluorescence in situ hybridization (HCR RNA-FISH) that *Anableps tbx5b* and *vax2* are expressed in the dorsal and ventral regions of the retina, respectively, during development, and that these expression domains are maintained in the adult retina of both *Anableps* and zebrafish (Supplementary Fig. 4).

**Fig. 4:**
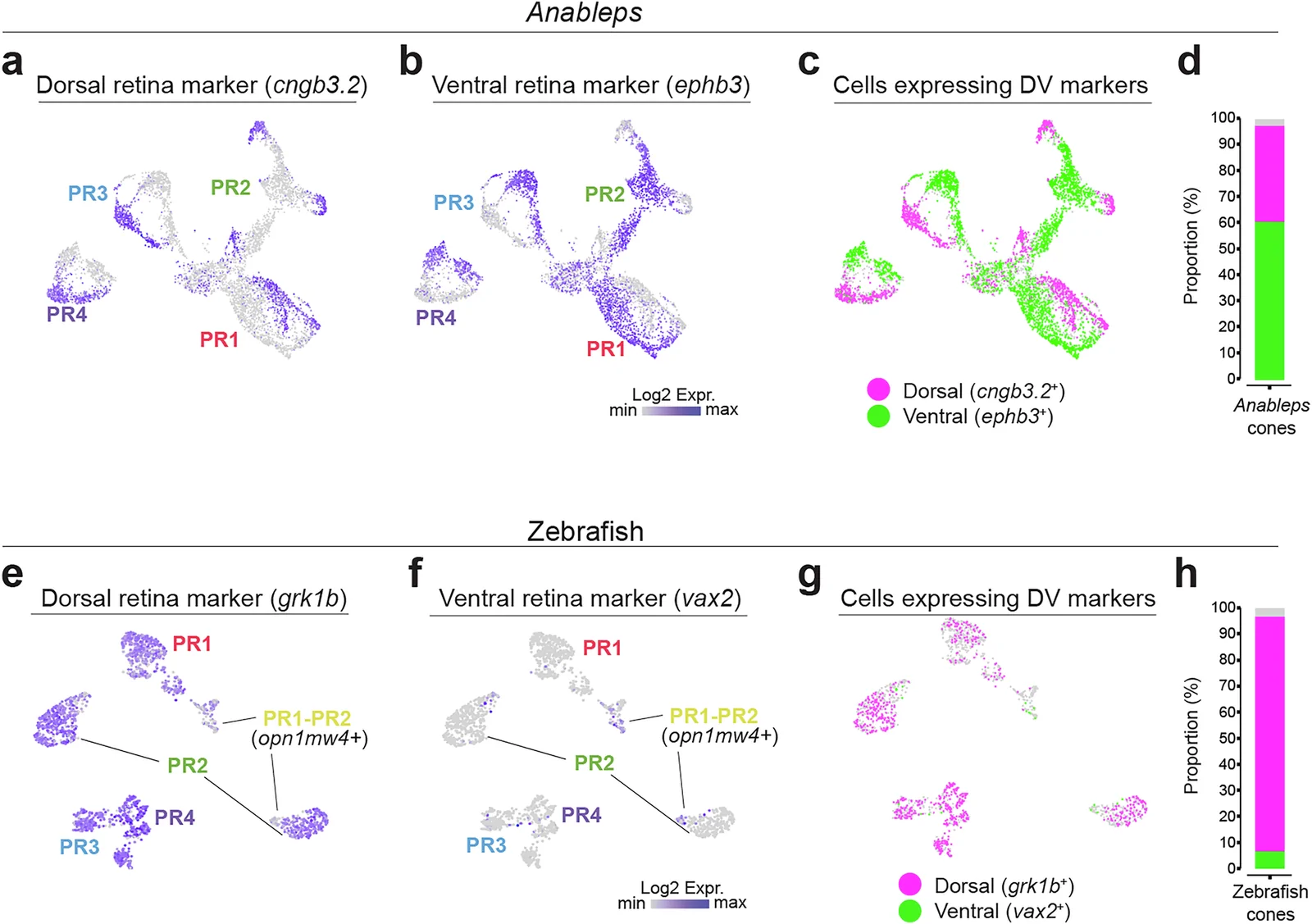
Expanded ventral representation of ancestral cone subtypes in *Anableps*. UMAP plots showing expression of *cngb3.2* and *grk1b* in the cone clusters of the *Anableps* (**a**) and zebrafish (**e**) retinas. UMAP plots showing expression of *ephb3* and *vax2* in the cone clusters of the *Anableps* (**b**) and zebrafish (**f**) retinas. UMAP plots highlighting dorsal (magenta) and ventral (green) cells identified by dorsal-ventral marker expression in the *Anableps* (**c**) and zebrafish (**g**) retinas; Bar plots showing the proportion of cells assigned to each retinal region based on dorsoventral marker expression (**d**) *Anableps* and (**h**) zebrafish.

To identify robustly expressed dorsal-ventral markers in *Anableps*, we searched for genes with the highest expression correlation with *tbx5b* and *vax2*. Among *tbx5b*-correlated genes, *cngb3.2*, which encodes the cyclic nucleotide-gated channel β3 subunit that account for ~50% of autosomal recessive achromatopsia cases in humans^15^, showed the strongest expression, over 10-fold greater than that of *tbx5b* (Supplementary Fig. 5a). Conversely, *ephb3*, a well-established ventral retina marker in vertebrates^16,17^, showed an expression level approximately 5-fold higher than *vax2* and was the most highly expressed gene correlated with *vax2* (Supplementary Fig. 5b). UMAP plots and bar graphic revealed *cngb3.2* enrichment in subsets of PR1, PR2, PR3, and PR4 cone clusters defining one subpopulation within each (Fig. 4a), whereas *ephb3* was enriched in complementary subpopulations (Fig. 4b). The two markers showed largely non-overlapping expression across cone clusters, with nearly 60% of *Anableps* cone cells expressing the ventral marker *ephb3* (Fig. 4c, d).

In the zebrafish scRNA-seq dataset, we used *grk1b* (a recently identified dorsal cone marker^8^) and *vax2* as dorsal and ventral markers, respectively. UMAP plots and bar graphic showed broad *grk1b* expression across PR1, PR2, PR3, and PR4 clusters, and partially in PR1-PR2 cone clusters (Fig. 4e). In contrast, *vax2* was enriched in PR1 cones and present in subsets of PR1-PR2 and PR2 clusters (Fig. 4f). UMAP plots confirmed largely exclusive expressions of *grk1b* and *vax2*, with *vax2*⁺ cells representing approximately 8% of all cones in the zebrafish retina (Fig. 4g, h and Supplementary Fig. 5d).

Together, these findings indicate that both dorsal and ventral regions of the *Anableps* retina contain the full complement of ancestral cone subtypes, PR1–PR4. In contrast, consistent with previous reports^8,18^, zebrafish show marked regional differences in the relative representation of PR1-PR4 cone subtypes, with ventral identity restricted to a smaller fraction of the cone population. These results suggest that ventral cone molecular identity is more broadly represented in the *Anableps* retina, while maintaining the full complement of ancestral cone subtypes. However, the ~60% and ~8% proportions were derived using different marker genes and sequencing modalities across species and should not be interpreted as precise, directly comparable quantitative measures.

### Regional opsin expression and predicted spectral tuning in *Anableps*

Our snRNA-seq dataset enabled classification of the ancestral cone subtypes and characterization of the opsins expressed by each subtype. In addition, analyses of dorsal-ventral patterning markers allowed us to assign cone populations dorsal or ventral retinal identity in both *Anableps* and zebrafish. Notably, *Anableps opn1mw2* and zebrafish *opn1mw4*, though members of the middle-wavelength-sensitive (MWS) opsin class, were both expressed in the ventral retina in *thrb*⁺ PR1 cones in *Anableps* and in ventrally enriched PR1-PR2 cones in the zebrafish (Fig. 3b, f). This suggests that these RH2-family opsins have undergone red-shifted spectral tuning relative to other middle-wavelength-sensitive paralogs. In *Anableps*, the red-shifted *opn1mw2* paralog is expressed in PR1 photoreceptors together with LWS opsins, potentially broadening their spectral sensitivity across middle-to-long wavelengths.

To estimate the peak wavelength sensitivity (*λ*_max_) of *Anableps* opsins, we applied OPTICS, a recently developed machine-learning framework that predicts photopigment absorbance spectra directly from opsin amino acid sequences^19^. OPTICS first extracts features related to spectral tuning from each opsin sequence, then uses a neural network trained on a broad dataset of vertebrate opsins with known *λ*_max_ values. The algorithm infers how variant amino acids drive shifts in wavelength sensitivity, enabling prediction of *λ*_max_ even in species where no experimental pigment reconstitution has been performed.

**Fig. 5:**
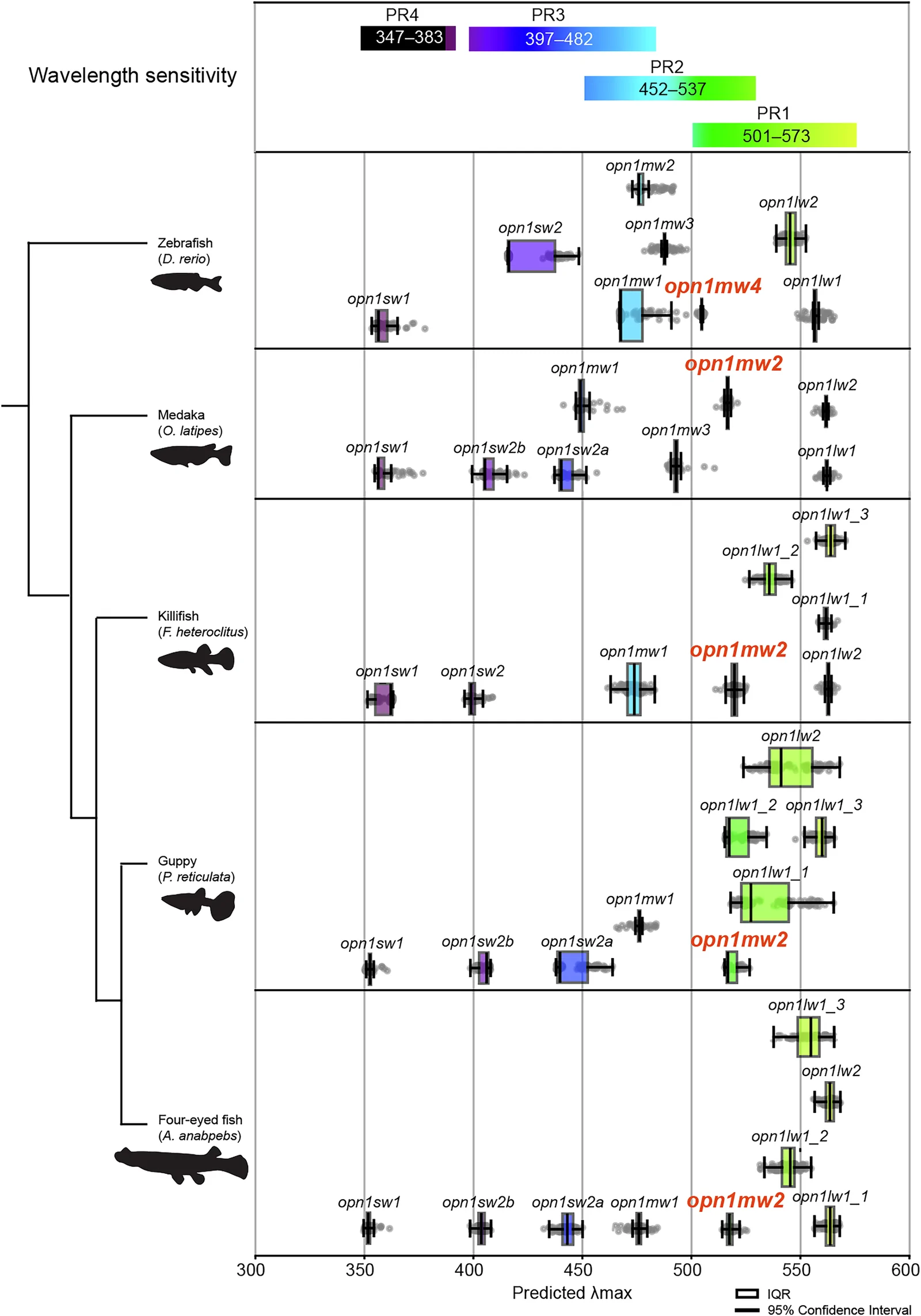
Predicted spectral tuning reveals a relative red shift of Opn1mw2 orthologs within the RH2 opsin family. Prediction of peak wavelength sensitivity (*λ*_max_) of visual opsins of zebrafish, medaka, killifish, guppy and *Anableps*. Opn1mw2 orthologs are red-shifted relative to other RH2 pigments but remain blue-shifted relative to the Opn1lw pigments expressed in dorsal *Anableps* PR1 cones. Boxplots show the distribution of predicted *λ*_max_ values from n = 100 bootstrap model predictions per opsin sequence (OPTICS v1.3, “wildtype-vert-mnm” model, default parameters), not biological or technical replicates; a single reference amino acid sequence per opsin per species was used as input. Box bounds indicate the interquartile range (IQR, 25–75th percentile); the center line indicates the median predicted *λ*_max_; whiskers indicate the 95% confidence interval (2.5th–97.5th percentile) of the bootstrap distribution; individual gray points represent predictions from individual bootstrap models.

To validate OPTICS predictions, we first analyzed zebrafish opsins whose *λ*_max_ values have been experimentally characterized for short-wavelength (*opn1sw1*: ~365 nm; *opn1sw2*: ~416 nm)^20^, middle-wavelength (*opn1mw1-4*: 467, 476, 488, 505 nm, respectively)^12^ and long-wavelength (*opn1lw1*: 558 nm; *opn1lw2*: 548 nm)^12^ pigments. OPTICS predictions closely matched these values (*opn1sw1*: 355 nm; *opn1sw2*: 416 nm; *opn1mw1-4*: 467, 475, 487, 505 nm; *opn1lw1*: 557 nm; *opn1lw2*: 545 nm) (Fig. 5; Supplementary data 2), demonstrating strong predictive accuracy.

Next, to explore the evolution of opsin spectral tuning in *Anableps*, we estimated *λ*_max_ values for visual opsins from medaka (*O. latipes*), killifish (*F. heteroclitus*), guppy (*P. reticulata*) and *Anableps*. Across these species, the most striking pattern was a red shift in Opn1mw2 orthologs relative to other RH2-family pigments. This shift predates the emergence of aerial vision in *Anableps*. The predicted *λ*_max_ of 517 nm indicates that *Anableps* Opn1mw2 extends PR1 cone sensitivity toward longer wavelengths relative to Opn1mw1, while remaining blue-shifted relative to the Opn1lw pigments expressed by dorsal PR1 cones.

### Dorsal extension of the ventral retina in *Anableps*

**Fig. 6:**
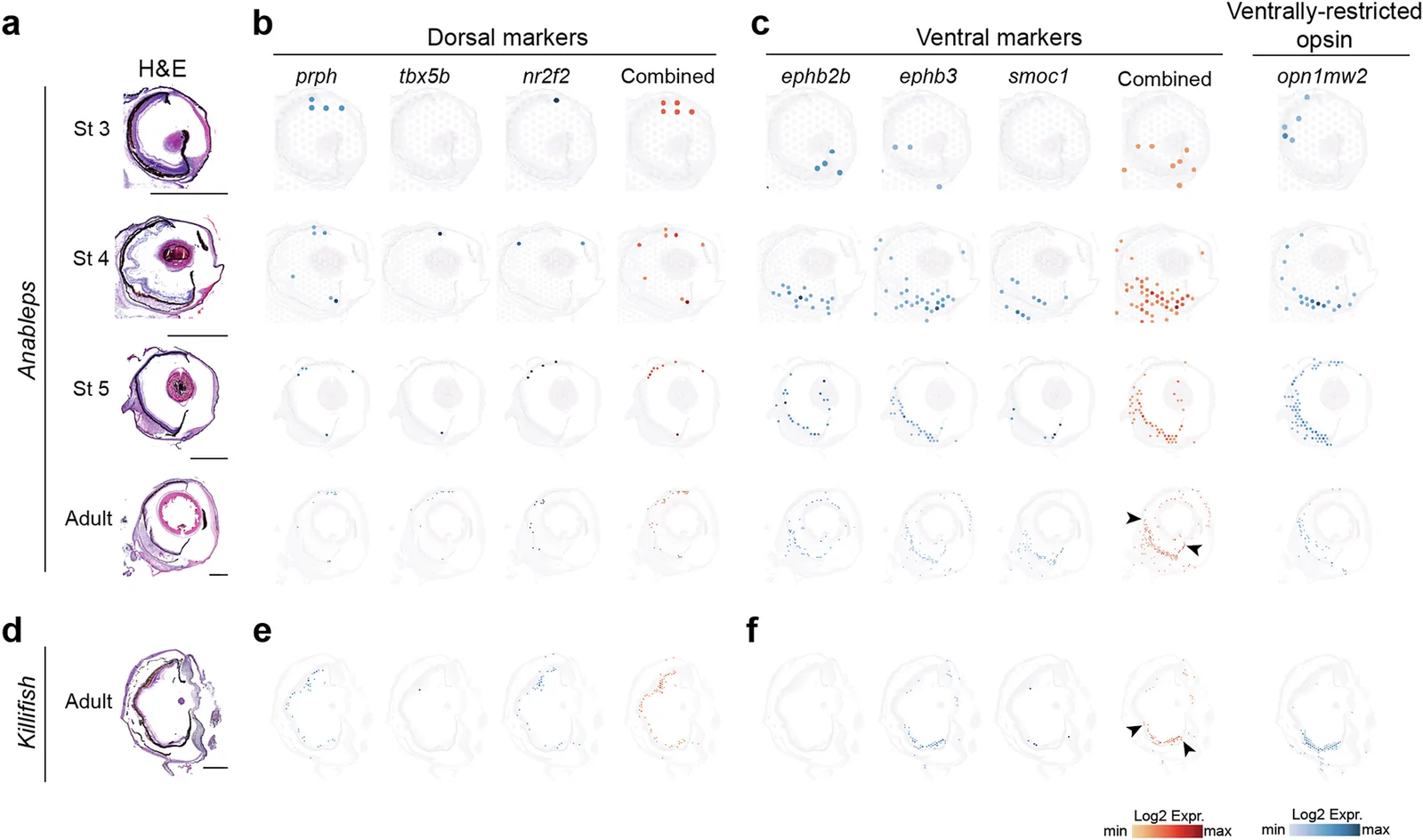
Comparative spatial transcriptomics of *Anableps* and killifish reveals dorsal expansion of the ventral retina. **a**,**d** Histological sections used for spatial transcriptomics. **b**,**c**,**e**,**f** Spatial RNA-seq showing expression of dorsal (**b**,**e**) and ventral (**c**,**f**) markers across developmental stages and adulthood in *Anableps* (**b**,**c**) and adult killifish (**e**,**f**). Combined expression of these markers and of the ventrally expressed *opn1mw2* gene highlights the expansion of the ventral retina in *Anableps* relative to killifish, indicated by black arrows in (**c**,**f**). Scale bar = 1 mm.

Previous reports^3,4^ and our current findings suggest that the ventral retina in *Anableps* has expanded in cell content relative to the dorsal retina. To examine the establishment of dorsal-ventral retinal territories during development and whether increased cell numbers correspond to spatial expansion, we performed spatial RNA-seq on developing *Anableps* eyes. Analysis of dorsal (*prph, tbx5b*, and *nr2f2*) and ventral (*ephb2b, ephb3*, and *smoc1*) marker expression across developmental stages 3–5, and in adults, revealed gradual regionalization of marker expression that persisted into adulthood (Fig. 6a–c). Combined expression of ventral markers indicated that the ventral retina ultimately occupies roughly half of the dorsal-ventral length of the *Anableps* retina (Fig. 6c, bottom right). Next, we assessed the same dorsal-ventral marker set in the adult killifish eye using spatial RNA-seq (Fig. 6d–f). We additionally examined expression of the killifish *opn1mw2* ortholog, which, like *Anableps opn1mw2*, was restricted to the ventralmost retina within the *ephb3*-positive domain (Fig. 6c, f). Despite this conserved ventral molecular identity, the ventral retina in killifish occupied only about one-fourth to one-third of the dorsal-ventral retinal length (Fig. 6f). These findings support the conclusion that the ventral retina expanded dorsally in *Anableps* sometime after the divergence of Anablepidae and Fundulidae.

## Discussion

Morphological innovations allow organisms to adapt to new niches and exploit new ecological opportunities, yet how such innovations arise has been a longstanding problem in evolutionary biology. A mechanistic understanding of the cellular and molecular bases of morphological innovations relies on organismal models amenable to experiments in a laboratory setting. Here we leveraged cutting-edge transcriptomic techniques to survey the *Anableps* retina at single-cell resolution and obtain a comprehensive, spatially resolved map of developing and adult retinas, and employed a machine-learning-based approach to evaluate the evolution of *Anableps* opsin gene sensitivity. Furthermore, our comparative analysis with an existing zebrafish scRNA-seq^8^ and our newly generated killifish spatial RNA-seq datasets provided evolutionary context to the unique adaptations that led to the remodeling of the *Anableps* ventral retina.

**Fig. 7:**
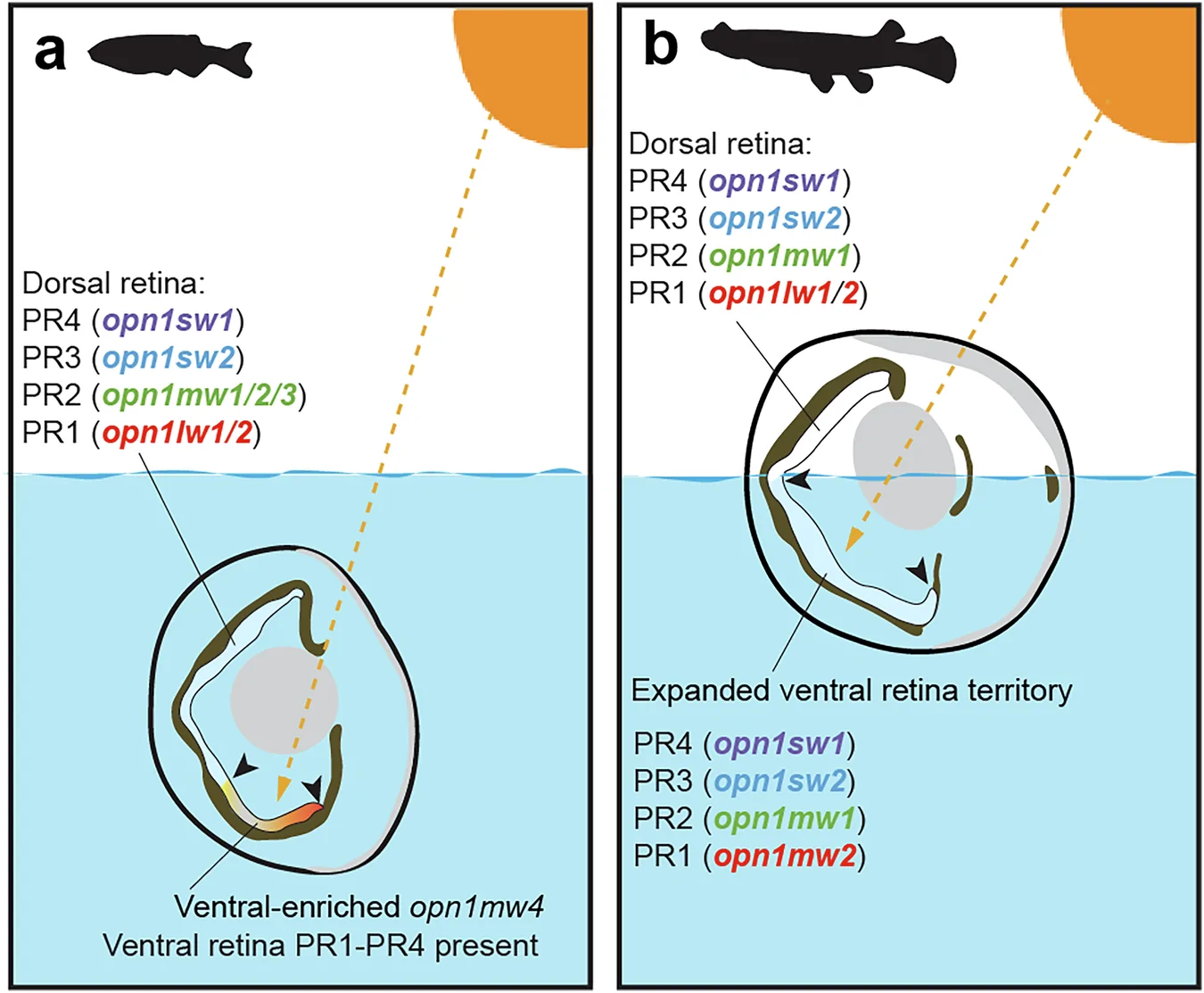
Expansion of the ventral retinal domain and regional redeployment of opsin expression underlie dual-environment vision in *Anableps*. **a**,**b** Schematic representation of dorsal and ventral retinal territories, PR1–PR4 cone subtype composition, and regional opsin expression in zebrafish (**a**) and *Anableps* (**b**) eye. Black arrowheads indicate the approximate region occupied by the ventral retina.

Aquatic teleost retinas commonly exhibit regional differences in cone abundance and opsin expression that are associated with variation in the underwater light environment. In zebrafish, the ventral retina is enriched for the red-shifted RH2 paralog *opn1mw4* and also contains *opn1lw1*-expressing PR1 cones, consistent with sensitivity extending toward longer wavelengths in the ventral visual field (Fig. 7a). In *Anableps*, the ventral retina instead expresses *opn1mw2* in PR1 cones, whereas the longer-wavelength-sensitive *opn1lw* paralogs are restricted dorsally (Fig. 7b).

*Anableps* occupies the air-water interface, and its ventral retina therefore receives direct aerial illumination and operates in an optical environment distinct from that encountered by the dorsal retina below the water surface. This ecological transition is accompanied by expansion of the ventral retinal territory and region-specific opsin expression within conserved cone subtypes. Our analysis showed that the *Anableps* ventral retina contains all four ancestral cone subtypes, but PR1 cells in this region express *opn1mw2* rather than the *opn1lw* paralogs expressed dorsally (Fig. 7b). OPTICS predicts that Opn1mw2 is red-shifted relative to Opn1mw1, suggesting that it extends the sensitivity of ventral PR1 cones toward longer wavelengths within the RH2 range. However, its predicted *λ*_max_ remains shorter than that of the dorsal Opn1lw pigments, and therefore it should not be considered functionally equivalent to an LWS opsin. The predicted red shift of Opn1mw2 is also present in medaka, killifish, and guppy, indicating that this spectral property predates the evolution of aerial vision in *Anableps*. The derived feature in *Anableps* is therefore not the origin of the red-shifted pigment itself, but its deployment in ventral PR1 cones within an expanded aerially specialized retinal domain.

Comparable regional and environmentally associated shifts in opsin expression have been described in several teleost lineages, including flounders and African cichlids^21–24^. These examples suggest that region-specific modulation of opsin deployment has evolved repeatedly in fishes. Whether the regulatory mechanisms underlying these shifts are shared across teleosts or have evolved independently remains unknown.

In addition to these region-specific changes in opsin deployment, the ventral retina of *Anablep*s samples the aerial visual field, and selection may therefore have favored expansion of this retinal territory together with increased ventral representation across multiple ancestral cone subtypes. Indeed, several anatomical and molecular observations support this notion: the ventral half of the *Anableps* retina contains approximately 3.6-fold more neurons in the ganglion cell layer than the dorsal half^10^, the ventral inner nuclear layer is roughly twice as thick, and *opn1mw2* expression is confined to the ventral retina with a sharp boundary at the dorsal-ventral midline^3^. Collectively, these findings point to a substantial enlargement and functional specialization of the ventral retinal territory in *Anableps*.

An unresolved question is the developmental origin of this expansion. The disproportionate ventralization of the retina could have arisen either through a true enlargement of ventrally specified territories during development or through functional reprogramming of regions that were originally dorsal in identity. To distinguish between these possibilities, we examined the expression of canonical dorsal-ventral markers during *Anableps* development and in the adult retina, alongside comparisons with the adult killifish. Our analyses show that the ventral half of the *Anableps* retina retains a ventral molecular identity, as indicated by persistent expression of ventral markers. Thus, aerial specialization involved expansion of a developmentally ventral retinal territory together with altered opsin deployment within conserved cone subtypes, rather than the evolution of new photoreceptor subtype identities. This interpretation is further supported by the expression of the killifish *opn1mw2* ortholog, which is likewise confined to the ventralmost region of the retina within the *ephb3*-positive domain.

Together, our findings illuminate how developmental patterning, region-specific opsin deployment, and expansion of a ventrally specified retinal domain were integrated to generate the distinctive visual system of *Anableps*. More broadly, these results exemplify how the modification of ancestral developmental programs can give rise to new sensory architectures and ecological capabilities, highlighting a general route by which morphological novelties emerge during vertebrate evolution.

## Methods

### Animal work

*Anableps* and zebrafish were maintained and used in accordance with approved Louisiana State University (LSU) IACUC protocols IACUCAM-25-094 and IACUCAM-24-003, respectively. Fish were obtained from commercial vendors. *Anableps* were maintained in colonies of 8–10 individuals in large tanks within a recirculating freshwater system at 27-28 °C under a 12 h light/12 h dark cycle. Zebrafish were kept in small tanks within a recirculating freshwater system at 27–28 °C under a 12 h light/12 h dark cycle. Prior to eye dissection, animals were euthanized with 0.3 % MS-222.

### Nuclei preparation and snRNA-seq sequencing

Five biological replicates of retinas from adult *Anableps* specimens were dissected and minced with a razor blade in a Petri dish on ice. The minced tissue was resuspended in 1 mL of lysis buffer (ATAC-seq lysis buffer, Active Motif Inc.) and then homogenized using a pre-chilled Dounce homogenizer (3 strokes). The homogenate was filtered through a 40 µm cell strainer and immediately centrifuged in an Eppendorf Protein LoBind tube at 500 x g at 4 °C for 5 min in a swinging bucket rotor. The pellet was quickly washed with 1 mL 1X PBS/0.5 % BSA and then resuspended in 1 mL of 1X PBS/0.5 % BSA. Nuclei were counted using a hemocytometer and then a volume corresponding to 10^6^ nuclei was transferred to a new tube and centrifuged at 500 x g at 4 °C for 5 min. Nuclei were fixed and frozen using the Evercode Nuclei Fixation Kit v3 (Parse Biosciences) following the manufacturer’s protocol. Five independent biological replicates were prepared. Fixed and frozen nuclei were then processed to generate eight RNA-sequencing libraries, using the Evercode^TM^ WT v3 Kit following the manufacturer’s protocol.

### SnRNA-seq processing and downstream analysis

SnRNA-seq FASTQ files were processed using the Trailmaker^TM^ piping module pipeline (https://app.trailmaker.parsebiosciences.com/; v1.4.0, Parse Biosciences, 2024). The data analysis and the heatmaps, dot plots, violin plots, and UMAP dimensionality reduction plots were generated using the Seurat v5 R package in R Studio and the TrailmakerTM platform.

We first corrected for ambient RNA contamination independently for each replicate using SoupX (v1.6.2)^25^, with estimated contamination fractions (ρ) ranging from 2.7% to 4.4%, and 1,203 putative doublets were removed via scds (v1.18.0; 2% expected rate)^26^. Replicate Seurat objects were generated from the corrected matrices, excluding features detected in fewer than 5 cells. Cells with fewer than 200 detected genes were discarded as empty droplets, and upper QC thresholds were set per sample based on gene and UMI quantile distributions. Mitochondrial filtering was omitted because mitochondrial genes were absent from the *Anableps anableps* reference genome annotation. Following initial filtering, a total of 53,998 high-quality nuclei were retained. Datasets were merged, normalized, and integrated using Harmony across 30 principal components. Louvain clustering at 0.5 resolution and UMAP visualization were applied. Finally, RStudio-based cluster filtering removed two rounds of low-quality clusters (immune contamination, smooth muscle cells, residual doublets, and batch artifacts) alongside the ‘RPE 3’ cluster, resulting in 47,586 nuclei across 23 annotated cell types (Fig. 1c and Supplementary Fig. 1).

To enable direct comparison with the *Anableps* dataset, the publicly available zebrafish scRNA-seq dataset^8^ was reprocessed from the raw FASTQ files using the same standardized computational workflow employed throughout this study, rather than relying on the original processed cell annotations. This approach ensured that cell filtering, normalization, clustering, and downstream analyses were performed using a consistent analytical framework across species. ScRNA-seq FASTQ files from zebrafish retina (GEO accession GSE175929)^8^ were reprocessed using Cell Ranger (10x Genomics), with cellranger mkref used to index the zebrafish reference genome (GRCz11) and cellranger count to map reads. The output matrices were imported into R and analyzed using Seurat (v5). Ambient RNA contamination was corrected using SoupX (v1.6.2)^25^ (rho = 0.032). Cells were filtered based on quality control thresholds (minimum 500 genes, maximum 40,000 UMI counts, and less than 30% mitochondrial reads). Data normalization was performed using SCTransform (glmGamPoi method, 3000 variable features), followed by PCA (30 components), Louvain clustering (resolution = 1.2), and UMAP visualization. Non-photoreceptor clusters, including Müller glia and doublet clusters were removed through two iterative filtering rounds, yielding a final dataset of 2,159 cells across 5 annotated cell types. After processing, the output files (barcodes.tsv.gz, features.tsv.gz, and matrix.mtx.gz) were uploaded to Trailmaker^TM^, where the subsequent analysis was performed.

To identify genes correlated with the dorsal marker *tbx5b* and ventral marker *vax2* in *Anableps*, and *grk1b* and v*ax2* in zebrafish, Pearson correlation coefficients (r) were calculated between the log-normalized expression of each reference marker and all other detected genes, computed per cell across subclustered cone cells using the cor() function in R. Genes were ranked by r, and the top three positively correlated genes (excluding the reference gene itself) were selected for visualization (Supplementary Fig. 5).

### Spatial RNA-seq library preparation and sequencing

Spatial transcriptomics was performed using the Visium Spatial Gene Expression platform (10x Genomics). The eyes of *Anableps* and killifish were processed following the Visium Spatial Tissue Preparation protocol from 10x Genomics. Briefly, *Anableps* (adult, stage 3, stage 4, and stage 5) and adult killifish were euthanized using 0.3 % MS-222, the eyes (adults and stage 5) were removed, and the heads (stage 3 and 4) were flash-frozen in Tissue-Tek Optimal Cutting Temperature (OCT) compound (Tissue Tek Sakura) using liquid nitrogen and isopentane, then stored at -80 °C. The tissues were cryosectioned at 10 µm at -20 °C using a Leica CM1520 cryostat and placed onto Visium Spatial Gene Expression slides (10x Genomics). The slides were fixed in cold methanol and stained with Hematoxylin and Eosin (H&E) (Sigma-Aldrich). Tissue permeabilization and reverse transcription (RT) were performed using the Visium Spatial Tissue Optimization Kit (10x Genomics), with a permeabilization time of 18 minutes. Images of H&E-stained sections and tissue optimization were captured using a Leica DM6B Fluorescence Microscope, equipped with a Leica DFC450 color CCD and Hamamatsu sCMOS camera, using a 20x objective. Gene expression slides and reagent kits were prepared according to the 10x Genomics protocol to produce sequencing libraries. The libraries were sequenced by Novogene Corporation Inc. on a NextSeq 6000 (Illumina) platform, generating 80 million paired-end reads of 150 bp and 50,000 transcripts per spot.

### Spatial transcriptomics data processing and downstream analysis

Gene expression quantification for the Visium dataset was performed using Space Ranger (version 2.1.1, 10x Genomics). The *Anableps* genome (GCA_014839685.1) and killifish genome (GCF_011125445.2) were indexed with mkref under Space Ranger. Subsequently, reads were aligned and quantified using Space Ranger count. Clustering and gene analysis were conducted using the Loupe Browser from the 10x Genomics Visium platform (version 8.1.2).

### Machine learning prediction of opsin spectral sensitivity

Maximum wavelength sensitivity of visual opsins was predicted using the Opsin Phenotype Tool for Inference of Color Sensitivity (OPTICS), a machine learning approach that infers maximum wavelength sensitive (*λ*_max_) directly from amino acid sequences^19^. Each opsin sequence was aligned to the wildtype-vert-mnm reference database using MAFFT within the optics_predictions.py pipeline, with bootstrapping and BLASTp analyses performed using bovine opsin as a reference. Bootstrapping and BLASTp analyses were performed using bovine opsin as a reference. Predictions were generated using the “wildtype-vert-mnm” model with amino-acid property (aa-prop) sequence encoding and default OPTICS parameters; bootstrap confidence intervals were derived from the default ensemble of n = 100 bootstrap models. Predicted *λ*_max_ values were compared across species to assess spectral tuning shifts and interpret cone subtype expression patterns in *Anableps*. Protein accession numbers for each opsin were as follows: *D. rerio*: *opn1sw1* (NP_571394.1), *opn1sw2* (NP_571267.1), *opn1mw4* (NP_571329.1), *opn1mw3* (NP_878312.1), *opn1mw2* (NP_878311.1), *opn1mw1* (NP_571328.2), *opn1lw1* (NP_001300644.1), and *opn1lw2* (NP_001002443.1). *O. latipes*: *opn1sw1* (NM_001104656.1), *opn1sw2-1* (XM_020703465.2), *opn1sw2-2* (NM_001104654.1), *opn1lw1* (XM_004069094.4), *opn1lw2* (NM_001104694.1), *opn1mw3* (XM_004086132.4), *opn1mw2* (XM_004086130.4), and *opn1mw1* (NM_001104655.1). *F. heteroclitus*: *opn1sw1* (XM_036149145.1), *opn1sw2* (XM_036151441.1), *opn1lw1-1* (XM_036151440.1), *opn1lw1-2* (XM_012854938.3), *opn1lw1-3* (XM_012869094.3), *opn1lw2* (XM_036151579.1), *opn1mw1* (XM_012872728.3), and *opn1mw2* (XM_012859940.3). *P. reticulata*: *opn1sw1* (NM_001297490.1), *opn1sw2-1* (NM_001297456.1), *opn1sw2-2* (NM_001297454.1), *opn1mw1* (NM_001297458.1), *opn1mw2* (NM_001297460.1), *opn1lw1-1* (NM_001297452.1), *opn1lw1-2* (XM_008410572.2), *opn1lw1-3* (XM_008397471.2), and *opn1lw2* (NM_001297450.1). *A. anableps*: *opn1sw1* (ENSABLT00000040448.1), *opn1sw2-1* (ENSABLT00000001542.1), *opn1sw2-2* (ENSABLT00000001545.1), *opn1mw2* (ENSABLT00000017435.1), *opn1mw1* (ENSABLT00000017442.1), *opn1lw1-1* (ENSABLT00000001559.1), *opn1lw1-2* (ENSABLT00000001564.1), *opn1lw2* (ENSABLT00000001632.1), and *opn1lw1-3* (ENSABLT00000032578.1).

### Cryosectioning

Dissected eyes from adult and developmental stages of *Anableps* and zebrafish were flash-frozen using isopentane cooled with liquid nitrogen. Isopentane was dispensed into a metal container and placed in liquid nitrogen to equilibrate for about 10 minutes. Tissues were embedded in Tissue-Tek O.C.T. compound (Sakura) using appropriate molds for 30 minutes. Using forceps, the molds were placed into the equilibrated isopentane until frozen. Samples were stored at -80 °C for sectioning. The tissues were cryosectioned at 10 µm (thickness) at −20 °C using a Leica CM1520 cryostat. The slides were stored at -80 °C until further use.

### HCR™ Gold RNA-FISH

Probes targeting*tbx5b* and *vax2* for HCR RNA-FISH were designed and synthesized as oligo pools by Molecular Instruments for zebrafish and *Anableps*. HCR was performed on tissue sections using the HCR™ Gold RNA-FISH kit (Molecular Instruments) according to the manufacturer’s instructions. Briefly, sections were fixed in ice-cold 4% paraformaldehyde for 15 min at 4 °C, dehydrated in 50%, 70%, and two changes of 100% ethanol (5 min each, room temperature), rinsed in 1× PBS, and surrounded with a hydrophobic barrier. Slides were pre-hybridized for 10 min at 37 °C in HCR™ HiFi Probe Hybridization Buffer, then incubated overnight at 37 °C with 50–100 µl probe solution (probes diluted to 100 µl per slide in HCR™ HiFi Probe Hybridization Buffer). Post-hybridization, slides were washed four times for 15 min each in pre-warmed 1× HCR™ HiFi Probe Wash Buffer, pre-amplified for 30 min in HCR™ Gold Amplifier Buffer, and incubated overnight at room temperature with 100 µl of amplification solution containing 2 µl each of H1 and H2 hairpins denatured at 95 °C for 90 s and cooled for 30 min. Finally, sections were washed four times for 15 min each in 1× HCR™ Gold Amplifier Wash Buffer, mounted in antifade medium (with optional DAPI), coverslipped, and stored at 4 °C protected from light until imaging.

### Reporting summary

Further information on research design is available in the Nature Portfolio Reporting Summary linked to this article.

## Supplementary information

Supplementary Information

Transparent Peer Review file

Reporting Summary

Description of Additional Supplementary Files

Supplementary Data 1

Supplementary Data 2

## Data Availability

Raw sequencing data for single-nucleus RNA-seq and spatial transcriptomics RNA-seq generated in this study have been deposited in the Gene Expression Omnibus (GEO) and are available at under accession numbers GSE306228 and GSE306418, respectively. Processed snRNA-seq data are available at the Broad Single Cell Portal https://singlecell.broadinstitute.org/single_cell/study/SCP3821 (SCP: SCP3821).

## Code availability

All code used in this study is available at our GitHub repository (https://github.com/lnperez90/Perez-et-al-2025) and has been archived with Zenodo at https://doi.org/10.5281/zenodo.22797441^27^.
