# Supplementary material for: Evolutionary remodeling of the ventral retina enables aerial vision in the four-eyed fish Anableps anableps
Source: Nat Commun. 2026 Oct 14;17:11619. doi: 10.1038/s41467-026-78775-4 (PMC13754780; doi:10.1038/s41467-026-78775-4)
Supplement: Supplementary file 1 [file 41467_2026_78775_MOESM1_ESM.pdf]

Supplementary Information

Evolutionary remodeling of the ventral retina enables aerial vision in the four-eyed fish *Anableps anableps*

Louise Perez[1,†], Lina Shi[1,†], Alyssa Daspit[1], Keyla N. Pruett[1], Carsyn Smith[1], Anna Prideaux[1], Josane F. Sousa[1] and Patricia N. Schneider[1]*.

[1]Department of Biological Sciences, Louisiana State University, Baton Rouge, LA, 70803.
[†]These authors contributed equally.

*Corresponding author
Email: pschneider@lsu.edu

Keywords: *Anableps*, evolution, eye, retina, opsin.

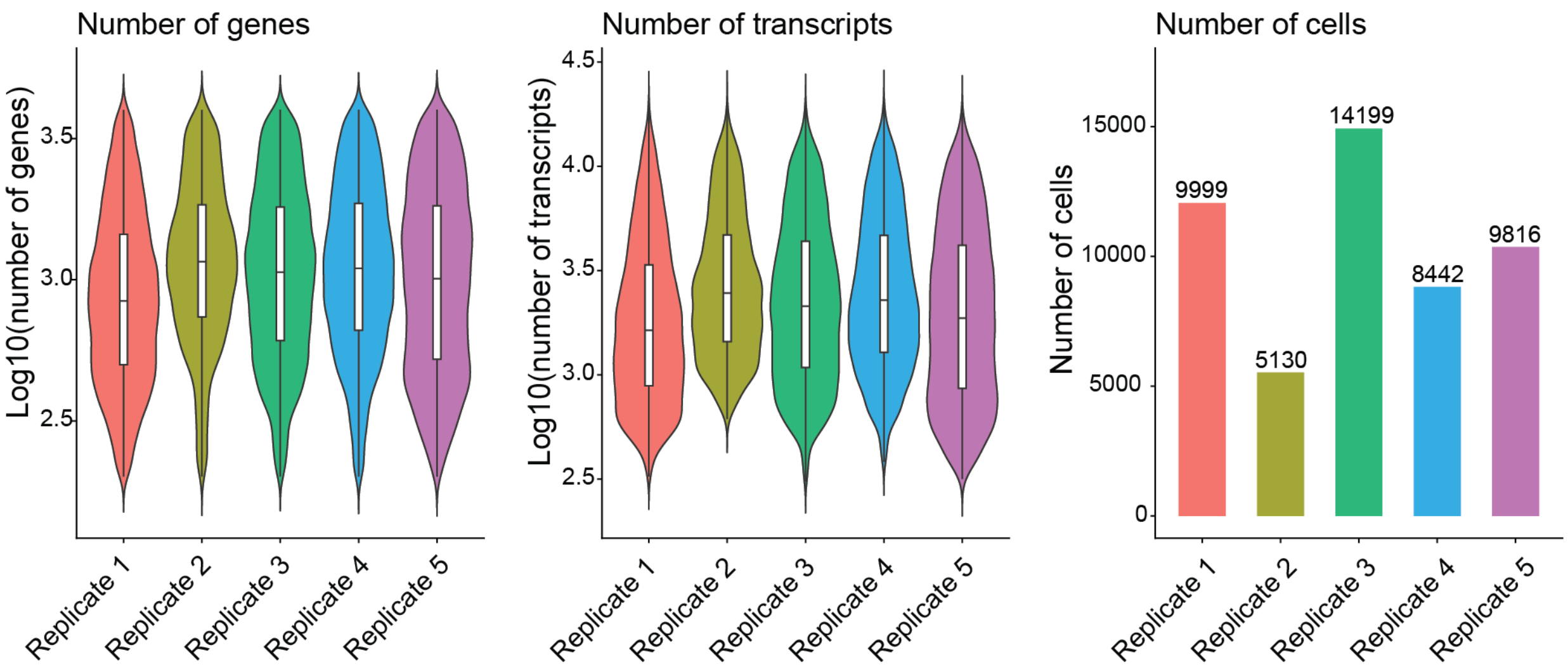

Supplementary Fig. 1. *Anableps* snRNA-seq quality control and marker gene expression across retinal cell clusters. QC metrics showing number of genes, transcripts, and cells (nuclei) across five independent biological replicates.

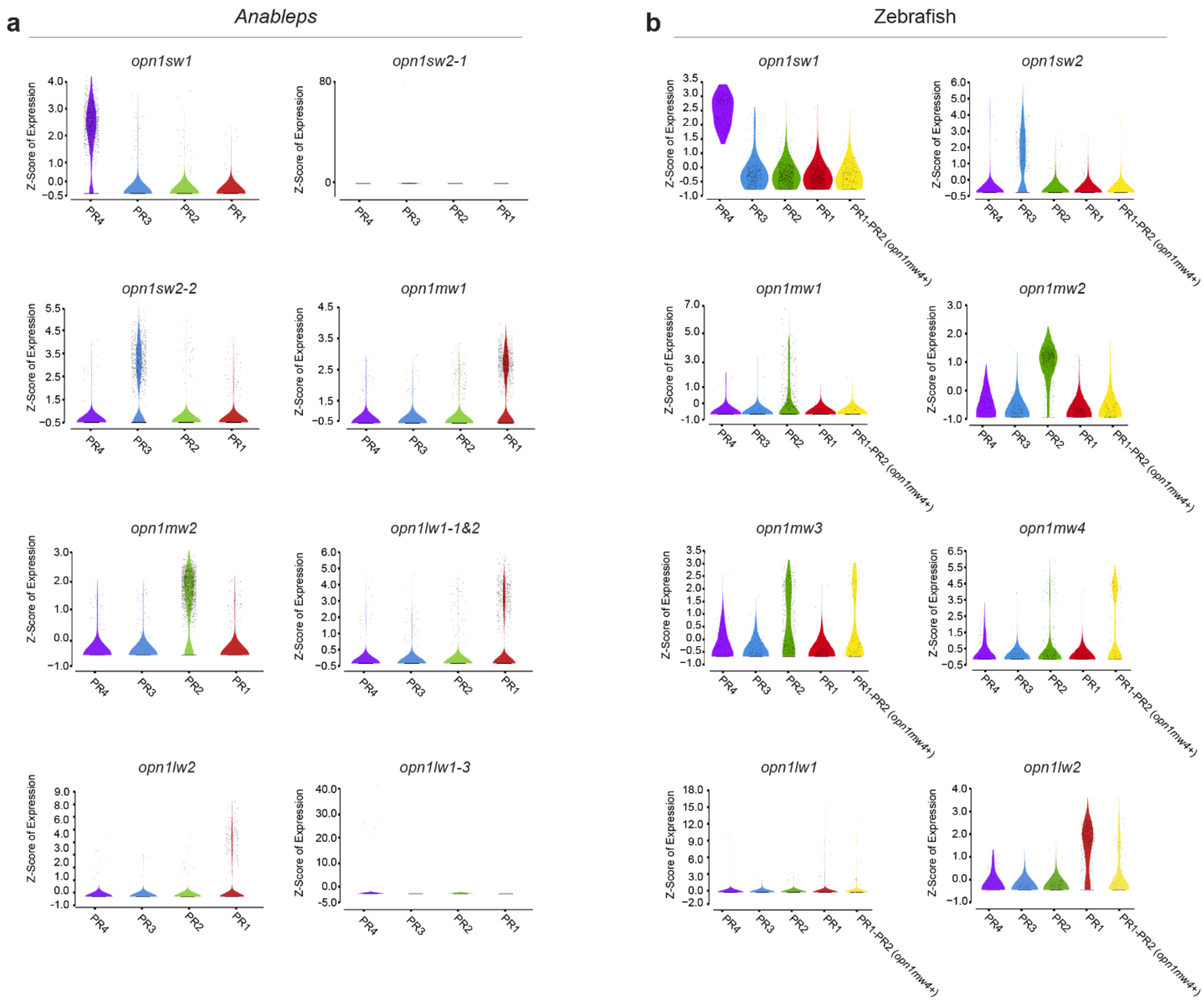

Supplementary Fig. 2. Visual opsin gene expression in cone clusters of *Anableps* and zebrafish. Violin plots show expression of visual opsin genes in the subclustered cone cells of the *Anableps* snRNA-seq (**a**) and zebrafish scRNA-seq (**b**) datasets.

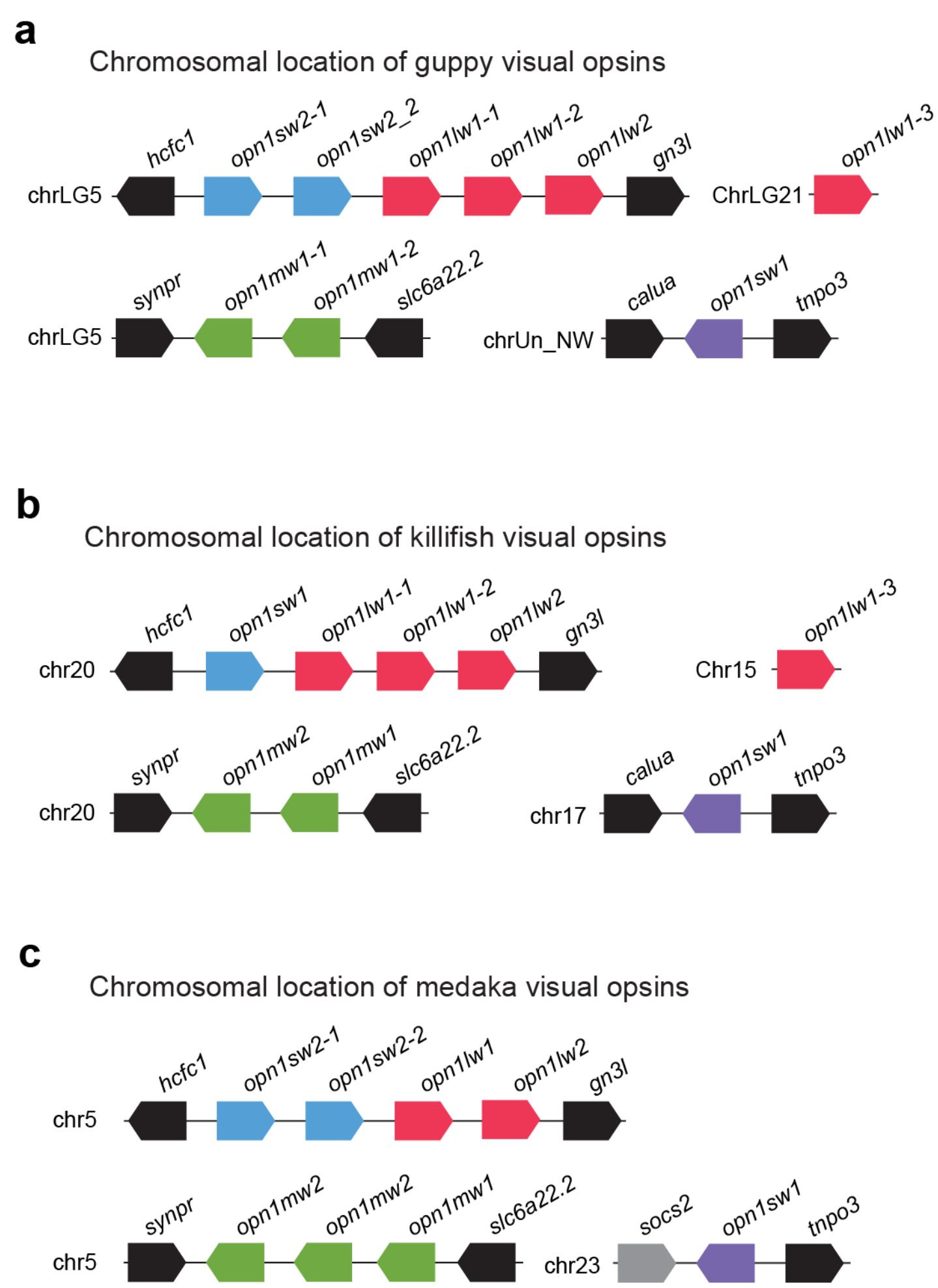

Supplementary Fig. 3. Gene organization of visual opsins in the genomes of guppy, killifish and medaka. Schematic drawings of genomic loci harboring visual opsin genes in guppy (**a**), killifish (**b**), and medaka (**c**).

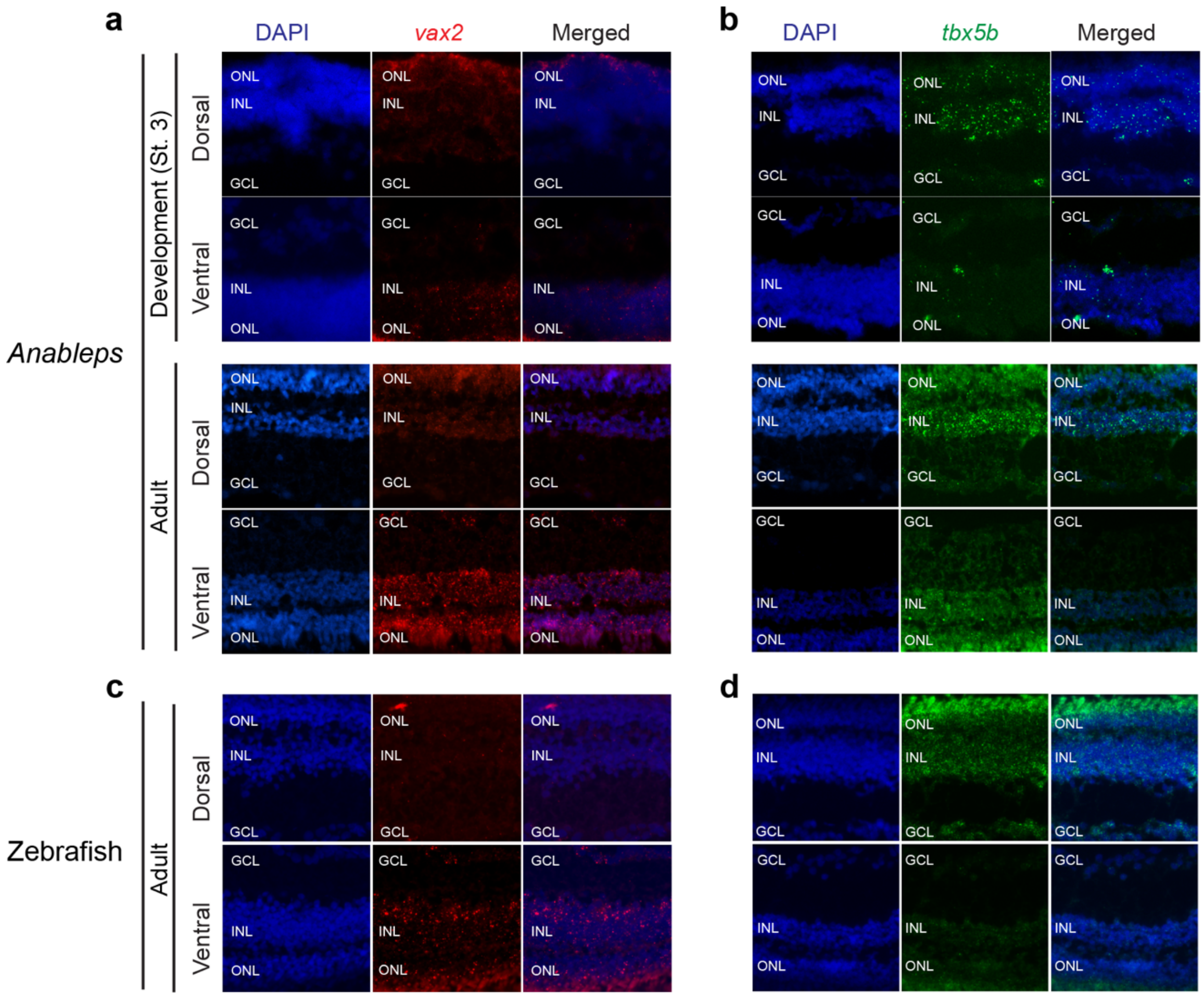

Supplementary Fig. 4. Expression of *tbx5b* and *vax2* orthologs in the dorsal and ventral retina regions of *Anableps* and zebrafish. a-**d**, HCR RNA-FISH showing expression of *vax2* (red) and *tbx5b* (green) in ventral and dorsal regions of the *Anableps* adult and stage 3 (**a**, **b**) and zebrafish (**c**, **d**) retinas. Nuclei were counterstained with DAPI (blue). ONL, outer nuclear layer; INL, inner nuclear layer; GCL, ganglion cell layer.

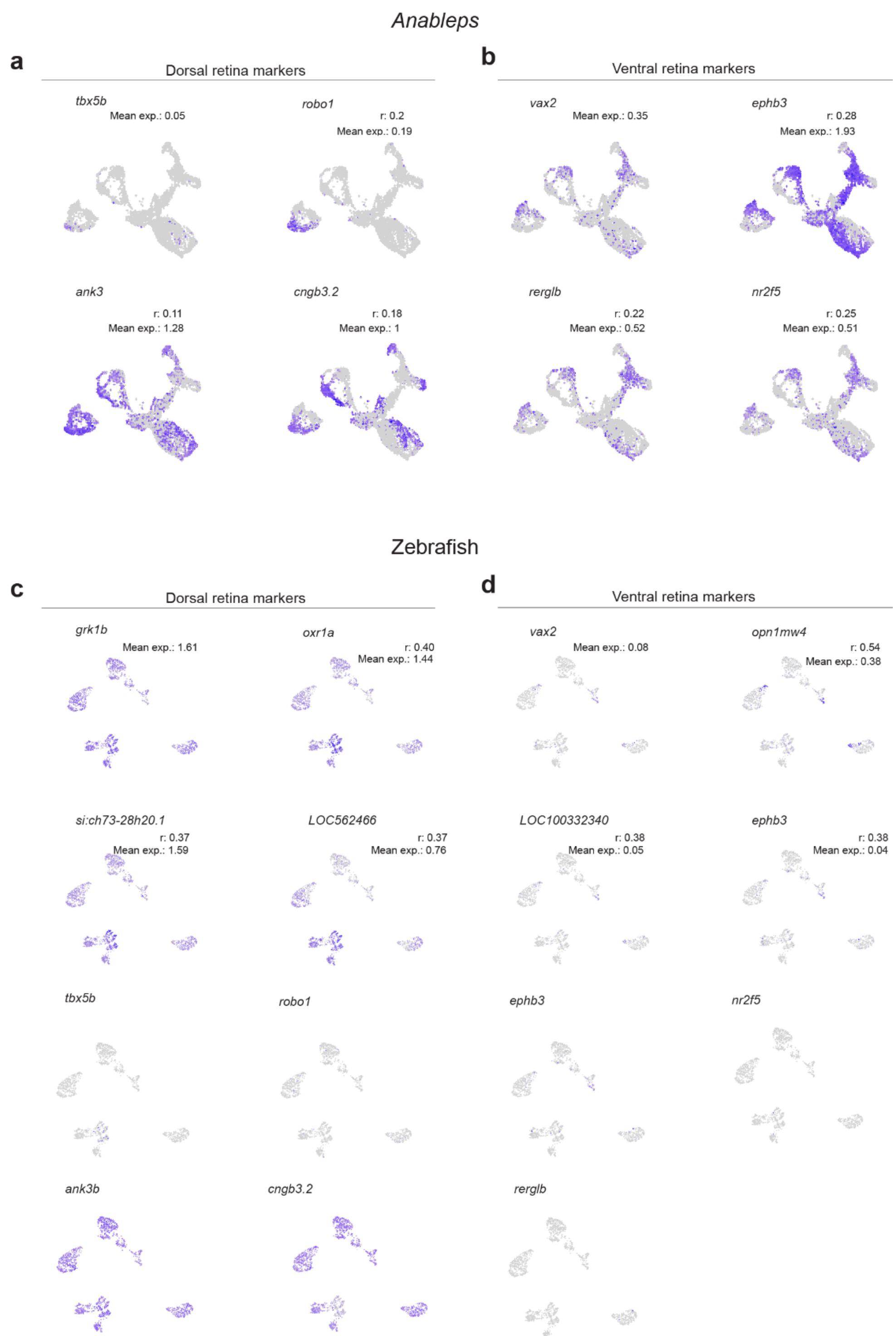

Supplementary Fig. 5. Expression of *tbx5b*, *vax2*, and correlated genes in *Anableps* and expression of *grk1b* and *vax2* in zebrafish cone cell clusters. UMAP plots showing expression of *tbx5b* (a) and *vax2* (b) and their three top correlated genes in cone cells in *Anableps*. UMAP plots showing expression of *grk1b* (c) and *vax2* (d) and their three top correlated genes in cone cells in zebrafish. The lower two rows of panels c and d show expression of the *Anableps*-derived dorsal (*tbx5b*, *robo1*, *ank3*, *cngb3.2*) and ventral (*ephb3*, *rerglb*, *nr2f5*) marker sets overlaid on the zebrafish dataset, assessing whether the same genes are similarly informative of dorsal-ventral identity across species. Mean exp., mean expression; r, correlation coefficient.
