# Supplementary material for: Evolutionary remodeling of the ventral retina enables aerial vision in the four-eyed fish Anableps anableps
Source: Nat Commun. 2026 Oct 14;17:11619. doi: 10.1038/s41467-026-78775-4 (PMC13754780; doi:10.1038/s41467-026-78775-4)
Supplement: Supplementary file 2 [file 41467_2026_78775_MOESM2_ESM.pdf]

nature portfolio

Peer Review File

Evolutionary remodeling of the ventral retina enables aerial vision in the four-eyed fish Anableps anableps

Corresponding Author: Professor Patricia Schneider

This file contains all reviewer reports in order by version, followed by all author rebuttals in order by version.

Version 0:

Reviewer comments:

Reviewer #1

(Remarks to the Author)
Perez et al. present a study on the retina of an unusual “four-eyed” fish species, Anableps anableps. This species doesn’t actually have four eyes (which should be clarified for readers) but swims with its eyes half out of water, so the ventral retina looks up through air while its dorsal retina looks down through the water.

Much of the paper is based on a single nucleus RNA sequencing analysis of the retina. Unfortunately, only a single biological sample was analyzed so there is no way to assess individual variations. Biological replicates are, at this point, standard in the field. In addition only 5668 high quality nuclei were obtained. Thus, there are far too few nuclei (cells) to cover even moderately abundant inner retinal cell types. The classes (amacrines, bipolars, etc.) are clearly separated, as verified by Visium platform spatial transcriptomics (Figure 2). Similarly, the the relatively abundant photoreceptor types seem to be well resolved as shown by assignment of opsins to clusters (Figure 3). However, based on studies of zebrafish, there are likely at least a dozen bipolar types and more than twice as many amacrine and retinal ganglion cell types, which are not separated here. Accordingly, this dataset is unlikely to be generally useful. This is a serious criticism, since previous studies have reported on the opsins (refs 3, 4, 9, 14 – but note, refs 3 and 9 are the same) so a main novelty of this paper is the description of the inner retina.

In a second set of studies, the authors ask whether the enlarged ventral domain, which mediates aerial vision “arose through expansion of ventrally specified cell types, or through functional reprogramming of developmentally dorsal retinal domains.” Using known markers (but not opsins), they provide evidence for the former alternative, then match opsin expression to the putative ventral and dorsal cells, showing differences among Anableps, zebrafish and killifish (Figures 4 and 6). This analysis also shows potential differences between the ventral and dorsal cohorts of red and blue opsins, but this is not followed up. To me, this is the strongest part of the paper. However, with the exception of fairly-low resolution images for one opsin in Figure 6, the distribution of the opsins is not mapped spatially, so key questions about the specialization of the ventral retina are left unanswered.

Finally, they use a machine learning method to estimate the spectral tuning of opsins in Anableps and four other fish species.

In summary, this work adds to our knowledge of Anableps vision, but may not provide decisive new insights that greatly extend our understanding of a fascinating and unusual retina.

Reviewer #2

(Remarks to the Author)
This is a new and exciting study from a group of researchers that has used Anableps to understand how the retina and visual system are modified to support the transition from aquatic to aerial vision. This is an interesting and unique model that is currently understudied and the authors keep providing important insights into how environmental cues produce adaptive changes in the eye, retina and visual system altogether. This research group has meticulously built a sequence of studies that together reveal how anableps fine-tunes its retinal opsin expression to support dual environment vision. They began by

characterizing opsin expression patterns in the dorsal and ventral retina of larval eyes from wild-caught females (Perez et al., 2017), establishing the foundation for understanding the species' split-retina organization. They then brought the animal into controlled laboratory conditions to test how juvenile anableps adjust opsin expression in response to different light environments (Salgado et al., 2022). Finally, in the current study, the authors here use cutting-edge technologies (single-nucleus and spatial transcriptomics and predictive machine learning) to reveal the cellular and molecular adaptations required for vision in aerial and aquatic environments. The authors describe interesting changes in photoreceptors and reveal mechanisms to partition the retina.
The study provides a novel and sound transcriptomic dataset from a unique fish species with a sub-functionalized eye, where the ventral retina specialises for aerial vision, while the ventral retina specialises for aquatic vision. The authors establish differences in gene expression in each domain and explore how opsin expression differences in photoreceptors lead to functional specialization for vision in each environment. The authors also identify unique transcriptional regulators that may drive this asymmetry. In summary, this study is relevant at the intersection of evolutionary biology, eye development and visual neuroscience and provide unique resources and important links to continue to understand the interplay between the environment and design of sensory systems.
The conclusions and claims are well supported by experimental results and we don't consider that additional experiments are required for publication. We also agree that the evidence provided in this study does support that "the Anableps ventral retina has been developmentally and functionally reconfigured for aerial vision". The methodology is sound: the use single nucleus and spatial transcriptomics are well supported and the experiments have been carried out well and to the standards of the field. The use of OPTICS to predict λmax is also well suited for the biological question and for interpretations. The majority of the methods are described in enough detail, and we appreciate that the dataset has been deposited in a public repository and can be easily and interactively explored in the Broad Institute's platform.
However, we have some disagreements on the wording of two of the proposed interpretations as stated here: "Interestingly, whereas the ventral retina of zebrafish contains cone types tuned to downwelling aquatic light, the Anableps ventral retina contains cones spanning all wavelength classes (UV, blue, green, and red), potentially enabling broad-spectrum sensitivity to aerial illumination."
This disagreement seems to be caused by the historical inconsistencies of the naming of photoreceptor subtypes. To avoid confusions between photoreceptor identity and opsin expression (as this study clearly exemplifies, these two are not equivalent), we will use the recently proposed standard nomenclature of vertebrate photoreceptor subtypes (10.1371/journal.pbio.3003157). Based on known evolutionary orthotypes and data presented here, both zebrafish and Anableps retain the ancestral set of cone subtypes:
PR1: referred here as Red, usually opn1lw-expressing and thrb+
PR2: referred here as Green, usually opn1mw-expressing and thrb-
PR3: referred here as Blue, usually opn1sw2-expressing and foxq2+
PR4: referred here as UV, usually opn1sw1-expressing and tbx2+
As stated by the authors, the findings in this study support the following model as the most likely for Anableps:

Dorsal retina:
- PR1: opn1lw1_1&2+ or opn1lw2+: λmax = 545 - 563 (nm)
- PR2: opn1mw1+: λmax = 476 (nm)
- PR3: opn1sw2_2+: λmax = 408 (nm)
- PR4: opn1sw1+: λmax = 350 (nm)

Ventral retina:
- PR1: opn1mw2+: λmax = 517 (nm)
- PR2: opn1mw1+: λmax = 476 (nm)
- PR3: opn1sw2_2+: λmax = 408 (nm)
- PR4: opn1sw1+: λmax = 350 (nm)

This implies that the ventral retina, which supports aerial vision, is less sensitive to long-wavelength light (especially above 545 nm) than the dorsal retina; in other words, sensitivity in the ventral retina is shifted towards shorter-wavelengths and has a narrower spectral sensitivity compared to the dorsal retina. In spite of Opn1mw2 being sensitive to longer wavelengths than Opn1mw1, we suggest that the authors reevaluate statements suggesting that the ventral/aerial PR1 cones are shifted towards longer wavelengths and a broader spectrum (line 107 and 259) and reassess the relation to the aerial and aquatic environments.

Regarding zebrafish, the authors seem to imply that the ventral zebrafish retina is only populated with opn1mw4- and opn1lw1-expressing cones (lines 101, 222, Figure 4E, and Figure 7), which is not accurate. This conclusion seems to stem from the fact that in the re-analysis of the Ogawa and Corbo dataset, the authors can't find ventral markers in many of the cone clusters (Figure 4E). Rather than the non-existence of PR2, PR3 and PR4 in the ventral retina, it is much more likely that the Ogawa and Corbo dataset is undersampled and the dataset does not contain a significant amount of ventral cones that would allow this analysis; this could be caused by biases in dissection, in cell capture through FACS, because of lack of sequencing depth or many other technical hurdles. In addition, the presence of PR2, PR3 and PR4 is thoroughly documented in the zebrafish retina. For example, PR4 in the ventro-temporal retina are known to be involved in larval prey hunting (Yoshimatsu et. al, 2020); subsequent work from this group shows larval recordings of activity for all cone subtypes across all retinal locations (Yoshimatsu et. al, 2020). The study from Zimmerman et. al, 2018, which is used to support this conclusion ("In contrast, zebrafish, consistent with previous reports, show a dorsal bias toward short-wavelength (UV/blue/green) cones") actually shows a different result, where PR4 are quite dense in the ventral retina, PR3 are densest in the central retina, and PR2 and PR1 have increased density in the nasal retina and in the dorso-temporal retina (Figure 2); in spite of these dense areas, the overall density of all the cone subtypes is quite high and there are no areas without any

of the cone subtypes (which would appear as white in the colored density maps). In adults, the presence of all photoreceptor subtypes in the ventral retina is clearly reported during the development of reporter lines (PR4: Takechi et. al, 2003; PR3: Takechi et. al, 2008; PR3: Tsujimura et. al, 2007), amongst many other studies. The authors should clearly state that "the ventral retina of zebrafish also contains all cone subtypes and spanning all wavelength classes".

We find that the comparisons between Anableps and zebrafish are useful and provide context to the results presented here. Nevertheless, in the Ogawa and Corbo study, the cluster that contains opn1mw4/opn1lw1-expressing cones are thrb+ (see Figure 1; this cluster is also positive for other well-characterised PR1 markers), but in the reanalysis of this dataset presented here, the authors find that this cluster of cones is thrb- (Figure 3F). The UMAP and clustering also look quite different from the original study, but there is no discussion on the discrepancies, if the reanalysis uncovered something novel or why reanalysis was required in the first place. It seems that the authors have split the opn1mw4/opn1lw1 cluster into two (as in Figure 2 of Ogawa and Corbo) but this is not explicit; moreover, after splitting there should still be ~50 opn1mw4+/opn1lw1- cells left, but this same cluster in Figure 3E seems to only contain a handful of cells in this new UMAP space? We recommend that the authors clarify these points and also to provide a way to replicate their RNAseq re-analysis to better support the species comparisons.
In any case, given the published results, the most likely model is that, in zebrafish, ventral PR4 express opn1lw1 (which has been confirmed in other studies) and ventral PR3 express opn1mw4, which indeed corresponds to shifts in sensitivity towards longer wavelengths compared to the dorsal retina's PR4 and PR3 respectively. The results presented here for Anableps stand in clear contrast to zebrafish. Interestingly, similar patterns have been described in other fish species, like flounders, cichlids and others (Savelli et al., 2018). In African cichlids, PR4 can express Rh2Aα (e.g. Spady et al., 2003), depending on species and depth (Ricci et al., 2023), and (as in Anableps) with dorso-ventral differences that are influenced by the environmental illumination (Dalton et al., 2016). Would the authors speculate if the same mechanisms may be at play (Sandkam et al., 2020) or if new regulators need to be identified?

Minor comments:
Figure 4: The legend of the figure (UMAP and dot plots) does not correspond to the panels (UMAP and violin plots).
Figure 4: Are the dorso ventral markers identified for Anableps also differentially expressed in the dorsal vs. ventral retina of zebrafish? Exposing this zebrafish data (as Supplementary 5b) would be informative.
Figure 4c & 4f: The legend for color coding is mislabeled:
Dorsal marker cngb3.2 became ventral (ephb3+)
Ventral retina marker ephb3 became Dorsal (cngb3.2+)
Dorsal marker grk1b became Dorsal (grk1b+) but the the corresponding area was labeled magenta
Ventral marker vax2 became Ventral (vax2+) but the corresponding area was labeled green.
Methods: In a previous study on anableps (Salgado et al., 2022), the authors showed that in wild juvenile Anableps moved from natural light and murky river to lab light and clear water, the expression of rh2-1 (opn1mw2) extended from ventral retina to both ventral and dorsal retina. The results presented here seem opposite, since samples collected were maintained in a lab environment (line 332)? Is there a simple explanation for this discrepancy?
Supplementary Figure 1a: What does each dot in these plots represent? A cell? If that's the case, wouldn't the close correspondence between number of transcripts and number of genes (~3000) mean that each cell has ~1 transcript for each gene?
Supplementary Figure 1a: Amacrine is mispelled
Supplementary Figure 2a: is opn1sw2_1 mislabeled as opn1sw1?
Supplementary file 1 is supposed to be top 100, but there's only 43 genes. Is sheet "_1" missing?
Reference 9, 11: html tags not correctly interpreted
Reference 21: DOI needs corrected

Reviewer #3

(Remarks to the Author)
I co-reviewed this manuscript with one of the reviewers who provided the listed reports. This is part of the Nature Communications initiative to facilitate training in peer review and to provide appropriate recognition for Early Career Researchers who co-review manuscripts.

Version 1:

Reviewer comments:

Reviewer #1

(Remarks to the Author)
My main criticism of the original manuscript was that there was only a single biological replicate of the single cell RNAseq data. The authors have now added four more, which fully satisfies my concern. The other changes are also valuable. I have no further criticisms or comments.

Reviewer #2

(Remarks to the Author)

The authors have substantially revised the manuscript in response to the previous reviews. The expanded snRNA-seq dataset, now including five biological replicates and 47,586 nuclei, which is a major improvement over the original dataset. The revised manuscript also makes an important conceptual distinction between photoreceptor identity and opsin expression. This improves the interpretation of the cone data. The revised manuscript now has the writing and experimental evidence to support the claim that Anableps retinas conserved cone identities but modified their regional opsin expression within an expanded ventrally specified retinal territory. The revised interpretation of Opn1mw2 is also more accurate, as the authors now describe it as relatively red-shifted within the Opn1mw family while remaining blue-shifted relative to Opn1lw opsin. The authors have addressed the majority of the reviewers concerns. Overall, we think this study remains an exciting and novel study to understand how the visual system and the retina are modified to support the transition from aquatic to aerial vision.

Some minor concerns remain:

- Photosensitive pigmented neurons: this is an undefined term that we find no previous use of in other studies, so we are unclear of what this cluster of cells is. "Pigmented" is conventionnally used for the melanin-containing retinal epithelium, but are authors refering to melanopsin-expressing ganglion cells? We advise using nomenclature with clear definitions.
- The use of HCR RNA-FISH to validate the dorsal and ventral markers, followed by identification of more robustly detected correlated markers in the snRNA-seq dataset, strengthens the dorsal-ventral classification. The observation that ventral molecular identity is represented in 60% of PR1-PR4 cones in Anableps is compelling. However, the quantitative comparison of ~60% ventral cones in Anableps with ~8% in zebrafish should be interpreted with caution, as these estimates are derived from different molecular classifiers and sequencing modalities (snRNA-seq vs scRNA-seq). These data support an increased representation of ventral molecular identity in Anableps, but they do not by themselves support the claim made in line 225 that there’s an increase in absolute cone cell number.
- Supplementary Fig. 1 refers to technical replicates, whereas the revised methods describe 5 biological replicates.
- Methods state that mitochondrial filtering was omitted because mitochondrial genes were absent from the reference genome annotation, whereas the legend to Supplementary Fig. 1 refers to the mitochondrial read fraction. Please clarify how this value was calculated.
- The post-mitotic neuroblast marker appears as crb2a+ in line 130 and crb2b+ in line 135.
- Figure 3F: revise gene nomenclature to match standard zebrafish gene names (e.g. opn1sw2)
bar graphic: bar graph?
- Supplementary Fig. 5 there are repeated plots and no description in methods on how correlation coefficients were calculated.
- This suggests that these RH2-family opsins have undergone red-shifted spectral tuning relative to other middle-wavelength-sensitive paralogs, extending the sensitivity of the corresponding ventral photoreceptors toward longer wavelengths.: this only hold if the red-shifted opsin was expressed by PR2 not PR1.

Open Access This Peer Review File is licensed under a Creative Commons Attribution 4.0 International License, which permits use, sharing, adaptation, distribution and reproduction in any medium or format, as long as you give appropriate credit to the original author(s) and the source, provide a link to the Creative Commons license, and indicate if changes were made.
In cases where reviewers are anonymous, credit should be given to 'Anonymous Referee' and the source.

The images or other third party material in this Peer Review File are included in the article's Creative Commons license, unless indicated otherwise in a credit line to the material. If material is not included in the article's Creative Commons license and your intended use is not permitted by statutory regulation or exceeds the permitted use, you will need to obtain permission directly from the copyright holder.
To view a copy of this license, visit https://creativecommons.org/licenses/by/4.0/

Reviewers' comments:

Reviewer #1 (Remarks to the Author):

Perez et al. present a study on the retina of an unusual "four-eyed" fish species, Anableps anableps. This species doesn't actually have four eyes (which should be clarified for readers) but swims with its eyes half out of water, so the ventral retina looks up through air while its dorsal retina looks down through the water.

Response: We thank the reviewer for this helpful suggestion. We agree that the common name "four-eyed fish" may be misleading to readers unfamiliar with *Anableps*. We have revised the Introduction to clarify that *Anableps anableps* possesses two eyes, each divided into dorsal and ventral optical compartments that simultaneously view aerial and aquatic environments.

Much of the paper is based on a single nucleus RNA sequencing analysis of the retina. Unfortunately, only a single biological sample was analyzed so there is no way to assess individual variations. Biological replicates are, at this point, standard in the field. In addition only 5668 high quality nuclei were obtained. Thus, there are far too few nuclei (cells) to cover even moderately abundant inner retinal cell types. The classes (amacrines, bipolars, etc.) are clearly separated, as verified by Visium platform spatial transcriptomics (Figure 2). Similarly, the the relatively abundant photoreceptor types seem to be well resolved as shown by assignment of opsins to clusters (Figure 3). However, based on studies of zebrafish, there are likely at least a dozen bipolar types and more than twice as many amacrine and retinal ganglion cell types, which are not separated here. Accordingly, this dataset is unlikely to be generally useful. This is a serious criticism, since previous studies have reported on the opsins (refs 3, 4, 9, 14 – but note, refs 3 and 9 are the same) so a main novelty of this paper is the description of the inner retina.

Response: We thank the reviewer for this thoughtful and constructive comment. We agree that the original snRNA-seq dataset was limited in both biological replication and sequencing depth. To address these concerns, we substantially expanded the single-nucleus analysis in the revised manuscript. The revised dataset now includes **five independent biological replicates** and **nearly 50,000 high-quality nuclei**, representing an approximately nine-fold increase in the number of nuclei analyzed compared to the original submission. This expanded dataset substantially improves the representation of retinal cell populations and enables improved resolution of neuronal subpopulations compared to the original version.

We also revised the manuscript to clarify that the goal of the single-nucleus analysis was not to generate a comprehensive cellular atlas of the *Anableps* retina. Rather, it provides the cellular and molecular framework necessary to investigate the developmental and evolutionary organization of the dorsal and ventral retina, particularly cone photoreceptor identity and dorsal-ventral retinal patterning, which form the basis for the subsequent spatial, developmental, and comparative analyses.

We agree that even deeper sequencing would likely resolve additional rare neuronal subtypes, including bipolar, amacrine, and retinal ganglion cells. Although comprehensive characterization of these populations lies beyond the scope of the present study, the substantially expanded dataset represents a valuable resource for the vision community. By making these data publicly available, future studies will be able to perform further subclustering analyses to refine and molecularly characterize these rarer retinal cell populations.

In a second set of studies, the authors ask whether the enlarged ventral domain, which mediates aerial vision "arose through expansion of ventrally specified cell types, or through functional reprogramming of developmentally dorsal retinal domains." Using known markers (but not opsins), they provide evidence for the former alternative, then match opsin expression to the putative ventral and dorsal cells, showing differences among Anableps, zebrafish and killifish (Figures 4 and 6). This analysis also shows potential differences between the ventral and dorsal cohorts of red and blue opsins, but this is not followed up. To me, this is the strongest part of the paper. However, with the exception of fairly-low resolution images for one opsin in Figure 6, the distribution of the opsins is not mapped spatially, so key questions about the specialization of the ventral retina are left unanswered.

Response: We thank the reviewer for this positive assessment of our study. We appreciate the reviewer's suggestion that additional spatial analyses of individual opsins could provide further anatomical detail regarding regional photoreceptor organization. However, the primary objective of this work was not to comprehensively remap the spatial distribution of visual opsins. The regional expression patterns of *Anableps* visual opsins have been described previously by our group and others (refs. 3 and 4), and we have revised the manuscript to better acknowledge these earlier studies. Rather than revisiting these established expression patterns, our goal was to integrate them with single-nucleus transcriptomics, spatial transcriptomics, and developmental analyses to address a previously unresolved evolutionary question: whether the enlarged ventral retina arose through expansion of a pre-existing ventral developmental domain or through reprogramming of dorsal retinal identity. To address this comment, we revised the manuscript to more clearly distinguish photoreceptor subtype identity from region-specific opsin expression throughout the Results and Discussion. We also incorporated additional comparative analyses showing

that the killifish *opn1mw2* ortholog is likewise restricted to the ventral *ephb3*-positive retinal domain. These findings support the conclusion that *Anableps* evolved aerial vision through expansion of a conserved ventral retinal territory rather than through the emergence of a novel retinal domain. While additional spatial analyses of individual opsins would undoubtedly provide additional anatomical detail, they would further refine the anatomical characterization of regional opsin expression, but would not alter the principal developmental conclusions of the present study.

Finally, they use a machine learning method to estimate the spectral tuning of opsins in Anableps and four other fish species.
In summary, this work adds to our knowledge of Anableps vision, but may not provide decisive new insights that greatly extend our understanding of a fascinating and unusual retina.

Response: We appreciate the reviewer's overall assessment. In the revised manuscript, we have substantially strengthened both the dataset and the framing of the study. The snRNA-seq analysis now includes five biological replicates and nearly 50,000 nuclei, and the revised manuscript more clearly emphasizes the central advance: identifying the developmental origin of the enlarged ventral retina in *Anableps*. By integrating single-nucleus and spatial transcriptomics across developmental stages and species, we show that this retinal specialization arose through dorsal expansion of a conserved ventral developmental territory together with region-specific opsin deployment within ancestral cone subtypes. We believe these findings extend previous descriptive studies of *Anableps* opsin expression by providing a developmental and cellular mechanism for the evolution of its dual visual system.

Reviewer #2 (Remarks to the Author):

This is a new and exciting study from a group of researchers that has used Anableps to understand how the retina and visual system are modified to support the transition from aquatic to aerial vision. This is an interesting and unique model that is currently understudied and the authors keep providing important insights into how environmental cues produce adaptive changes in the eye, retina and visual system altogether. This research group has meticulously built a sequence of studies that together reveal how anableps fine-tunes its retinal opsin expression to support dual environment vision. They began by characterizing opsin expression patterns in the dorsal and ventral retina of larval eyes from wild-caught females (Perez et al., 2017), establishing the foundation for understanding the species' split-retina organization. They then brought the animal into controlled laboratory conditions to test how juvenile anableps adjust opsin expression in response to different light environments (Salgado et al., 2022). Finally, in the current study, the authors here use cutting-edge technologies (single-nucleus and spatial transcriptomics and predictive machine learning) to reveal the cellular and molecular adaptations required for vision in aerial and aquatic environments. The authors describe interesting changes in photoreceptors and reveal mechanisms to partition the retina.
The study provides a novel and sound transcriptomic dataset from a unique fish species with a sub-functionalized eye, where the ventral retina specialises for aerial vision, while the ventral retina specialises for aquatic vision. The authors establish differences in gene expression in each domain and explore how opsin expression differences in photoreceptors lead to functional specialization for vision in each environment. The authors also identify unique transcriptional regulators that may drive this asymmetry. In summary, this study is relevant at the intersection of evolutionary biology, eye development and visual neuroscience and provide unique resources and important links to continue to understand the interplay between the environment and design of sensory systems.
The conclusions and claims are well supported by experimental results and we don't consider that additional experiments are required for publication. We also agree that the evidence provided in this study does support that "the Anableps ventral retina has been developmentally and functionally reconfigured for aerial vision". The methodology is sound: the use single nucleus and spatial transcriptomics are well supported and the experiments have been carried out well and to the standards of the field. The use of OPTICS to predict λmax is also well suited for the biological question and for interpretations. The majority of the methods are described in enough detail, and we appreciate that the dataset has been deposited in a public repository and can be easily and interactively explored in the Broad Institute's platform.

Response: We sincerely thank the reviewer for their thoughtful, detailed, and highly constructive evaluation of our work. We are particularly grateful for their positive assessment of the study's significance, methodology, and overall conclusions, and for recognizing the value of *Anableps* as a model for understanding the evolution of aerial and aquatic vision. We also appreciate the reviewer's careful consideration of the manuscript and the many insightful suggestions, which have helped us improve both the clarity and interpretation of our findings. We address each of the reviewer's comments below.

However, we have some disagreements on the wording of two of the proposed interpretations as stated here:
"Interestingly, whereas the ventral retina of zebrafish contains cone types tuned to downwelling aquatic light, the Anableps ventral retina contains cones spanning all wavelength classes (UV, blue, green, and red), potentially

enabling broad-spectrum sensitivity to aerial illumination."
This disagreement seems to be caused by the historical inconsistencies of the naming of photoreceptor subtypes. To avoid confusions between photoreceptor identity and opsin expression (as this study clearly exemplifies, these two are not equivalent), we will use the recently proposed standard nomenclature of vertebrate photoreceptor subtypes (10.1371/journal.pbio.3003157). Based on known evolutionary orthotypes and data presented here, both zebrafish and Anableps retain the ancestral set of cone subtypes:
PR1: referred here as Red, usually opn1lw-expressing and thrb+
PR2: referred here as Green, usually opn1mw-expressing and thrb-
PR3: referred here as Blue, usually opn1sw2-expressing and foxq2+
PR4: referred here as UV, usually opn1sw1-expressing and tbx2+
As stated by the authors, the findings in this study support the following model as the most likely for Anableps:

Dorsal retina:
- PR1: opn1lw1_1&2+ or opn1lw2+: λmax = 545 - 563 (nm)
- PR2: opn1mw1+: λmax = 476 (nm)
- PR3: opn1sw2_2+: λmax = 408 (nm)
- PR4: opn1sw1+: λmax = 350 (nm)

Ventral retina:
- PR1: opn1mw2+: λmax = 517 (nm)
- PR2: opn1mw1+: λmax = 476 (nm)
- PR3: opn1sw2_2+: λmax = 408 (nm)
- PR4: opn1sw1+: λmax = 350 (nm)

This implies that the ventral retina, which supports aerial vision, is less sensitive to long-wavelength light (especially above 545 nm) than the dorsal retina; in other words, sensitivity in the ventral retina is shifted towards shorter-wavelengths and has a narrower spectral sensitivity compared to the dorsal retina. In spite of Opn1mw2 being sensitive to longer wavelengths than Opn1mw1, we suggest that the authors reevaluate statements suggesting that the ventral/aerial PR1 cones are shifted towards longer wavelengths and a broader spectrum (line 107 and 259) and reassess the relation to the aerial and aquatic environments.

Response: We thank the reviewer for this thoughtful and insightful comment. We agree that our original wording did not consistently distinguish photoreceptor subtype identity from opsin expression, and that this distinction is particularly important when interpreting regional differences in spectral sensitivity. We appreciate the reviewer for highlighting the recently proposed standardized nomenclature for vertebrate cone photoreceptor subtypes, which provides a clearer framework for interpreting our findings.

In response to this comment, we have revised the manuscript throughout to adopt the PR1-PR4 nomenclature and to consistently distinguish cone subtype identity from the opsins expressed by those subtypes. We have also revised the Abstract, Introduction, Results, Discussion, and figure legends to avoid conflating photoreceptor subtype identity with spectral sensitivity.

We further agree that our previous wording overstated the interpretation of the ventral retina as exhibiting broader spectral sensitivity or a shift toward longer wavelengths. Accordingly, we have revised these statements to reflect the conclusions directly supported by our data. The revised manuscript now emphasizes that the *Anableps* ventral retina retains the full complement of ancestral cone subtypes while exhibiting region-specific opsin expression. Specifically, we now describe ventral PR1 cones as expressing the red-shifted RH2-family opsin *opn1mw2* rather than the longer-wavelength-sensitive *opn1lw* paralogs expressed dorsally, and clarify that the predicted spectral properties of Opn1mw2 remain blue-shifted relative to dorsal Opn1lw pigments.

Finally, we have revised the Discussion to emphasize that the principal finding of this study is not a global shift in spectral sensitivity, but rather that aerial vision evolved through developmental expansion of a conserved ventral retinal territory together with region-specific deployment of opsin expression within ancestral cone subtypes. We believe these revisions substantially improve the accuracy of our interpretation and are grateful to the reviewer for helping us clarify this important conceptual distinction.

Regarding zebrafish, the authors seem to imply that the ventral zebrafish retina is only populated with opn1mw4- and opn1lw1-expressing cones (lines 101, 222, Figure 4E, and Figure 7), which is not accurate. This conclusion seems to stem from the fact that in the re-analysis of the Ogawa and Corbo dataset, the authors can't find ventral markers in many of the cone clusters (Figure 4E). Rather than the non-existence of PR2, PR3 and PR4 in the ventral retina, it is much more likely that the Ogawa and Corbo dataset is undersampled and the dataset does not contain a significant amount of ventral cones that would allow this analysis; this could be caused by biases in dissection, in cell capture through FACS, because of lack of sequencing depth or many other technical hurdles. In addition, the presence of PR2, PR3 and PR4 is thoroughly documented in the zebrafish retina. For example, PR4 in the ventro-temporal retina are known to be involved in larval prey hunting (Yoshimatsu et. al, 2020); subsequent work from this group shows

larval recordings of activity for all cone subtypes across all retinal locations (Yoshimatsu et. al, 2020). The study from Zimmerman et. al, 2018, which is used to support this conclusion ("In contrast, zebrafish, consistent with previous reports, show a dorsal bias toward short-wavelength (UV/blue/green) cones") actually shows a different result, where PR4 are quite dense in the ventral retina, PR3 are densest in the central retina, and PR2 and PR1 have increased density in the nasal retina and in the dorso-temporal retina (Figure 2); in spite of these dense areas, the overall density of all the cone subtypes is quite high and there are no areas without any of the cone subtypes (which would appear as white in the colored density maps). In adults, the presence of all photoreceptor subtypes in the ventral retina is clearly reported during the development of reporter lines (PR4: Takechi et. al, 2003; PR3: Takechi et. al, 2008; PR3: Tsujimura et. al, 2007), amongst many other studies. The authors should clearly state that "the ventral retina of zebrafish also contains all cone subtypes and spanning all wavelength classes".

Response: We thank the reviewer for this important clarification and for highlighting the extensive literature describing the distribution of cone photoreceptor subtypes in the zebrafish retina. We agree that our original wording could be interpreted as implying that certain cone subtypes are absent from the ventral zebrafish retina, which was not our intention. Rather, our reanalysis detected expression of the ventral retina markers in only a subset of cone clusters within the published dataset, and we agree that this should not be interpreted as evidence that other cone subtypes are absent from the ventral retina.

In response to this comment, we have revised the manuscript throughout to avoid this interpretation. We now explicitly state that both zebrafish and *Anableps* retain the full complement of ancestral cone photoreceptor subtypes (PR1-PR4) in the ventral retina, consistent with previous anatomical and functional studies. We further clarify that the limited detection of ventral marker expression in our reanalysis most likely reflects the limited representation of ventral retinal cells in the published dataset rather than the true cellular composition of the zebrafish ventral retina.

Accordingly, we have revised the corresponding sections of the Abstract, Results, Discussion, and the schematic model (Fig. 7) so that the comparison between zebrafish and *Anableps* focuses on differences in regional opsin deployment and the developmental expansion of ventral retinal identity in *Anableps*, rather than on the presence or absence of particular cone subtypes. We believe these revisions more accurately reflect both the published zebrafish literature and the conclusions supported by our comparative analyses.
.

We find that the comparisons between Anableps and zebrafish are useful and provide context to the results presented here. Nevertheless, in the Ogawa and Corbo study, the cluster that contains opn1mw4/opn1lw1-expressing cones are thrb+ (see Figure 1; this cluster is also positive for other well-characterised PR1 markers), but in the reanalysis of this dataset presented here, the authors find that this cluster of cones is thrb- (Figure 3F). The UMAP and clustering also look quite different from the original study, but there is no discussion on the discrepancies, if the reanalysis uncovered something novel or why reanalysis was required in the first place. It seems that the authors have split the opn1mw4/opn1lw1 cluster into two (as in Figure 2 of Ogawa and Corbo) but this is not explicit; moreover, after splitting there should still be ~50 opn1mw4+/opn1lw1- cells left, but this same cluster in Figure 3E seems to only contain a handful of cells in this new UMAP space? We recommend that the authors clarify these points and also to provide a way to replicate their RNAseq re-analysis to better support the species comparisons. In any case, given the published results, the most likely model is that, in zebrafish, ventral PR4 express opn1lw1 (which has been confirmed in other studies) and ventral PR3 express opn1mw4, which indeed corresponds to shifts in sensitivity towards longer wavelengths compared to the dorsal retina's PR4 and PR3 respectively. The results presented here for Anableps stand in clear contrast to zebrafish. Interestingly, similar patterns have been described in other fish species, like flounders, cichlids and others (Savelli et al., 2018). In African cichlids, PR4 can express Rh2Aα (e.g. Spady et al., 2003), depending on species and depth (Ricci et al., 2023), and (as in Anableps) with dorso-ventral differences that are influenced by the environmental illumination (Dalton et al., 2016). Would the authors speculate if the same mechanisms may be at play (Sandkam et al., 2020) or if new regulators need to be identified?

Response: We thank the reviewer for carefully examining our reanalysis of the Ogawa and Corbo dataset and for identifying these points requiring clarification. We reanalyzed the raw zebrafish data to place the zebrafish and *Anableps* cone datasets within a comparable analytical framework and to examine the same subtype markers, opsins, and dorsal-ventral markers across species. Our intent was not to reproduce the exact UMAP presented in the original publication or to propose a revised biological classification of the zebrafish cone populations.

We agree that the differences between the two analyses should have been explained more clearly. Ogawa and Corbo used a custom adult-eye transcript annotation and a highly curated workflow involving deliberate overclustering followed by multiple rounds of cluster filtering and manual selection, ultimately retaining 2,186 photoreceptor and bipolar cells. In our analysis, reads were aligned to the GRCz11 reference using Cell Ranger and processed using the computational workflow employed throughout this study. Differences in reference annotation, normalization, quality-control thresholds, cell retention, and clustering are expected to contribute to differences in UMAP configuration and in the representation of the small ventral double-cone population.

We also agree that the weak or absent detection of *thrb* in this population in our reanalysis should not be interpreted as evidence that the *opn1lw1*-expressing cells lack PR1 identity. Consistent with the original Ogawa and Corbo annotation and the corresponding opsin expression patterns, we now describe this population as a ventrally enriched PR1-PR2 double-cone population containing *opn1lw1*-expressing PR1 cells and *opn1mw4*-expressing PR2 cells. We have revised the text and figure annotations accordingly and no longer use the lack of detectable *thrb* in our reanalysis to infer photoreceptor subtype identity.

To improve transparency and reproducibility, we have revised the Methods to explicitly explain why the zebrafish dataset was reanalyzed and to describe the computational workflow used for reprocessing the raw FASTQ files, including the quality-control, normalization, clustering, and downstream analytical steps.

We further appreciate the reviewer's suggestion to place our findings within a broader evolutionary context. Accordingly, we have expanded the Discussion to highlight comparable region-specific and environmentally associated shifts in opsin expression reported in other teleost lineages, including flounders and African cichlids. We also note that whether the regulatory mechanisms underlying these convergent patterns are shared across teleosts or have evolved independently remains unknown. We believe this places the *Anableps* findings within a broader comparative framework while emphasizing an important avenue for future investigation.

Minor comments:
Figure 4: The legend of the figure (UMAP and dot plots) does not correspond to the panels (UMAP and violin plots).

Response: Fixed.

Figure 4: Are the dorso ventral markers identified for Anableps also differentially expressed in the dorsal vs. ventral retina of zebrafish? Exposing this zebrafish data (as Supplementary 5b) would be informative.

Response: we have now expanded this figure. It includes markers identified via correlation with known DV zebrafish markers. (*grk1b*/vax2), but also the UMAP plots for the DV markers identified in *Anableps*. Most recapitulate the DV pattern seen for *grk1b*/vax2, some show little to no detectable expression in the zebrafish single cell dataset.

Figure 4c & 4f: The legend for color coding is mislabeled:
Dorsal marker cngb3.2 became ventral (ephb3+)
Ventral retina marker ephb3 became Dorsal (cngb3.2+)
Dorsal marker grk1b became Dorsal (grk1b+) but the the corresponding area was labeled magenta
Ventral marker vax2 became Ventral (vax2+) but the corresponding area was labeled green.

Response: Fixed.

Methods: In a previous study on anableps (Salgado et al., 2022), the authors showed that in wild juvenile Anableps moved from natural light and murky river to lab light and clear water, the expression of rh2-1 (opn1mw2) extended from ventral retina to both ventral and dorsal retina. The results presented here seem opposite, since samples collected were maintained in a lab environment (line 332)? Is there a simple explanation for this discrepancy?

Response: We thank the reviewer for raising this thoughtful point. We agree that our previous study demonstrated environmentally induced plasticity of *opn1mw2* (*rh2-1*) expression following transfer of juvenile *Anableps* from their natural murky-water habitat to laboratory aquaria, where dorsal expansion of *opn1mw2* expression was observed after 30 days. At first glance, this appears to differ from the ventrally restricted expression observed in the present study.

We believe, however, that these studies address different biological questions. The previous work specifically investigated the plasticity of opsin expression following environmental manipulation using RNA in situ hybridization, whereas the present study uses single-nucleus and spatial transcriptomics to define the developmental organization of retinal cell populations and dorsal-ventral retinal identity. Importantly, our principal conclusions do not depend solely on the expression domain of *opn1mw2*, but on the concordant spatial distribution of multiple independent ventral markers (e.g., *ephb2b*, *ephb3*, and *smoc1*) across development and adulthood, which consistently demonstrate expansion of a ventrally specified retinal territory.

At present, we do not believe there is a single definitive explanation for the apparent difference between the two studies. Several non-mutually exclusive factors may contribute, including differences in developmental stage, environmental history and lighting conditions, methodological differences between RNA in situ hybridization and transcriptomic profiling, or the possibility that environmentally induced changes in opsin expression represent transient plastic responses that are not maintained throughout life. It is also important to note that the environmental conditions in the previous study were not identical to those used for the animals analyzed here. In the earlier experiments, aquaria received substantial natural daylight in addition to artificial illumination, whereas the present colony was maintained under standard laboratory husbandry conditions. Although these differences were not the

focus of either study and cannot fully account for the observed discrepancy, they indicate that the lighting environments were not directly comparable. Determining the relative contribution of these factors will require dedicated longitudinal and functional studies and is beyond the scope of the present work.

Supplementary Figure 1a: What does each dot in these plots represent? A cell? If that's the case, wouldn't the close correspondence between number of transcripts and number of genes (~3000) mean that each cell has ~1 transcript for each gene?

Response: We thank the reviewer for raising this point. Yes, in the original Supplementary Figure 1, each dot represents an individual nucleus (used as a proxy for a cell). Please note that in the revised manuscript, Supplementary Figure 1a has been updated to reflect the expanded snRNA-seq dataset, which now includes 47,586 nuclei from five biological replicates.

The number of genes indicates the total number of unique genes detected per nucleus, whereas the number of transcripts refers to the total number of captured transcript molecules (UMIs) detected per nucleus, rather than transcript abundance or normalized gene expression. Importantly, the y-axes in these panels (as in the original figure) show log10-transformed values, not raw counts. Because of the compressive nature of a logarithmic scale, values that appear visually close can correspond to substantially different underlying numbers. Based on the medians shown in the updated figure, nuclei contain approximately 800–1,100 detected genes (log10 ≈ 2.9-3.05) and 1,600-2,500 detected transcript molecules (UMIs) (log10 ≈ 3.2-3.4), depending on the biological replicate. This corresponds to approximately 1.8-2.2 detected transcript molecules per detected gene, which is within the expected range for UMI-based single-cell/single-nucleus RNA-seq datasets, including those generated using the Parse Biosciences platform (Filippov et al., 2024).

Supplementary Figure 1a: Amacrine is misspelled

Response: Heatmap containing the typo has been removed as it was redundant to the dot plot on Figure 1.

Supplementary Figure 2a: is opn1sw2_1 mislabeled as opn1sw1?

Response: Fixed.

Supplementary file 1 is supposed to be top 100, but there's only 43 genes. Is sheet "_1" missing?

Response: original table was indeed incomplete. Current version is now corrected.

Reference 9, 11: html tags not correctly interpreted

Response: Fixed.

Reference 21: DOI needs corrected

Response: Fixed.

Reviewer #3 (Remarks to the Author):

I co-reviewed this manuscript with one of the reviewers who provided the listed reports. This is part of the Nature Communications initiative to facilitate training in peer review and to provide appropriate recognition for Early Career Researchers who co-review manuscripts.

RESPONSE TO REVIEWERS' COMMENTS

Reviewer #1 (Remarks to the Author):

My main criticism of the original manuscript was that there was only a single biological replicate of the single cell RNAseq data. The authors have now added four more, which fully satisfies my concern. The other changes are also valuable. I have no further criticisms or comments.

Reviewer #2 (Remarks to the Author):

The authors have substantially revised the manuscript in response to the previous reviews. The expanded snRNA-seq dataset, now including five biological replicates and 47,586 nuclei, which is a major improvement over the original dataset. The revised manuscript also makes an important conceptual distinction between photoreceptor identity and opsin expression. This improves the interpretation of the cone data. The revised manuscript now has the writing and experimental evidence to support the claim that Anableps retinas conserved cone identities but modified their regional opsin expression within an expanded ventrally specified retinal territory. The revised interpretation of Opn1mw2 is also more accurate, as the authors now describe it as relatively red-shifted within the Opn1mw family while remaining blue-shifted relative to Opn1lw opsin. The authors have addressed the majority of the reviewers concerns. Overall, we think this study remains an exciting and novel study to understand how the visual system and the retina are modified to support the transition from aquatic to aerial vision.

Some minor concerns remain:

- Photosensitive pigmented neurons: this is an undefined term that we find no previous use of in other studies, so we are unclear of what this cluster of cells is. "Pigmented" is conventionally used for the melanin-containing retinal epithelium, but are authors referring to melanopsin-expressing ganglion cells? We advise using nomenclature with clear definitions.

Response: We thank the reviewer for this comment. Our original designation was influenced by the striking enrichment of *opn5* in this population. Upon re-examination, *opn5* expression is robust and highly specific to this cluster (~88% of cells versus ~9% elsewhere), making an artifactual signal unlikely. The broader transcriptional profile strongly supports a pigment-cell identity, with canonical melanogenic markers (*pmela*, *tyrp1b*, *tyrp1*, *mlpha*, *oca2*, *mitfa*, and *kita*) co-localizing precisely with *opn5* expression in this cluster. The cluster also lies adjacent to the RPE populations in the UMAP. We therefore agree that "neuron" was not warranted and have renamed this population from "photosensitive pigmented neuron" to "*opn5*+ melanogenic pigment cell" throughout the manuscript and figures. This designation reflects its well-supported cellular identity while highlighting its distinctive *opn5* expression.

- The use of HCR RNA-FISH to validate the dorsal and ventral markers, followed by identification of more robustly detected correlated markers in the snRNA-seq dataset, strengthens the dorsal-ventral

classification. The observation that ventral molecular identity is represented in 60% of PR1-PR4 cones in Anableps is compelling. However, the quantitative comparison of ~60% ventral cones in Anableps with ~8% in zebrafish should be interpreted with caution, as these estimates are derived from different molecular classifiers and sequencing modalities (snRNA-seq vs scRNA-seq). These data support an increased representation of ventral molecular identity in Anableps, but they do not by themselves support the claim made in line 225 that there's an increase in absolute cone cell number.

Response: We thank the reviewer for this careful assessment and for the comments regarding the HCR RNA-FISH validation and correlation-based marker identification. We agree with both points raised. First, the ~60% (*Anableps*) vs. ~8% (zebrafish) comparison relies on different marker genes (*ephb3* vs. *vax2*) and sequencing modalities (snRNA-seq vs. scRNA-seq) and should not be interpreted as a precise, directly comparable quantitative measure across species. Second, we agree that the proportion of cones expressing ventral molecular identity reflects the relative composition of the sampled population and does not, on its own, establish an increase in absolute cone cell number. We have revised the sentence at line 225 accordingly: "These results suggest that ventral cone molecular identity is more broadly represented in the *Anableps* retina, while maintaining the full complement of ancestral cone subtypes. However, the ~60% and ~8% proportions were derived using different marker genes and sequencing modalities across species and should not be interpreted as precise, directly comparable quantitative measures."

- Supplementary Fig. 1 refers to technical replicates, whereas the revised methods describe 5 biological replicates.
- Methods state that mitochondrial filtering was omitted because mitochondrial genes were absent from the reference genome annotation, whereas the legend to Supplementary Fig. 1 refers to the mitochondrial read fraction. Please clarify how this value was calculated.

Response: We thank the reviewer for pointing this out. The reference to the mitochondrial read fraction in the legend of Supplementary Fig. 1 was an error. As mentioned in the main text, mitochondrial genes are absent from the *Anablep*s reference genome annotation; therefore, mitochondrial read filtering was not performed, and no mitochondrial read fraction was calculated. We have removed the mitochondrial read fraction from the legend of Supplementary Fig. 1 and revised the corresponding text in the Methods to clarify this point. Here is the revised legend: "Supplementary Fig. 1. *Anableps* snRNA-seq quality control and marker gene expression across retinal cell clusters. QC metrics showing the number of genes, transcripts, and cells (nuclei) across the five independent biological replicates."

- The post-mitotic neuroblast marker appears as crb2a+ in line 130 and crb2b+ in line 135.

We thank the reviewer for catching this mistake. This issue was fixed and they're now labeled correctly as crb2b+.

- Figure 3F: revise gene nomenclature to match standard zebrafish gene names (e.g. opn1sw2)
bar graphic: bar graph?

Response: We thank the reviewer for this comment. We have revised the gene nomenclature in Figure 3f to follow the standard zebrafish nomenclature where applicable while retaining the appropriate gene names for the additional *Anableps* paralogs. We have also revised the figure legend accordingly.

- Supplementary Fig. 5 there are repeated plots and no description in methods on how correlation coefficients were calculated.

Response: We thank the reviewer for identifying these issues. We have added a description of the correlation methodology to the Methods section: "To identify genes correlated with the dorsal marker *tbx5b* and ventral marker *vax2* in *Anableps*, and *grk1b* and v*ax2* in zebrafish, Pearson correlation coefficients (r) were calculated between the log-normalized expression of each reference marker and all other detected genes, computed per cell across subclustered cone cells using the cor() function in R. Genes were ranked by r, and the top three positively correlated genes (excluding the reference gene itself) were selected for visualization (Supplementary Fig. 5)."

We have also revised the legend to clarify that the lower rows of panels c and d show the *Anableps*-derived marker set plotted on the zebrafish dataset as a cross-species comparison, rather than a repeat of panels a/b. "Supplementary Fig. 5. Expression of *tbx5b*, *vax2*, and correlated genes in *Anableps* and expression of *grk1b* and *vax2* in zebrafish cone cell clusters. UMAP plots showing expression of *tbx5b* (a) and *vax2* (b) and their three top correlated genes in cone cells in *Anableps*. UMAP plots showing expression of *grk1b* (c) and *vax2* (d) and their three top correlated genes in cone cells in zebrafish. The lower two rows of panels c and d show expression of the *Anableps*-derived dorsal (*tbx5b*, *robo1*, *ank3*, *cngb3.2*) and ventral (*ephb3*, *rerglb*, *nr2f5*) marker sets overlaid on the zebrafish dataset, assessing whether the same genes are similarly informative of dorsal-ventral identity across species. Mean exp., mean expression; r, correlation coefficient".

In addition, we removed the repeated *vax2* in Supplementary fig. 5d.

- This suggests that these RH2-family opsins have undergone red-shifted spectral tuning relative to other middle-wavelength-sensitive paralogs, extending the sensitivity of the corresponding ventral photoreceptors toward longer wavelengths.: this only hold if the red-shifted opsin was expressed by PR2 not PR1.

Response: We thank the reviewer for pointing out that our wording was unclear. We agree that expression of the red-shifted *opn1mw2* paralog would not extend the spectral sensitivity of PR2 photoreceptors, as this paralog is instead expressed in PR1. Our intended point was that the predicted red shift of *opn1mw2* may contribute to broadening the spectral sensitivity of the PR1 cells in which it is expressed. We have amended the sentence to clarify this distinction:

"This suggests that these RH2-family opsins have undergone red-shifted spectral tuning relative to other middle-wavelength-sensitive paralogs. In *Anableps*, the red-shifted *opn1mw2* paralog is expressed in PR1 photoreceptors together with LWS opsins, potentially broadening their spectral sensitivity across middle-to-long wavelengths."
