# Supplementary material for: Evolutionary remodeling of the ventral retina enables aerial vision in the four-eyed fish Anableps anableps
Source: Nat Commun. 2026 Oct 14;17:11619. doi: 10.1038/s41467-026-78775-4 (PMC13754780; doi:10.1038/s41467-026-78775-4)
Supplement: Supplementary file 3 [file 41467_2026_78775_MOESM3_ESM.pdf]

nature portfolio

Corresponding author(s): Patricia Schneider

Last updated by author(s): 08/03/2026

Reporting Summary

Nature Portfolio wishes to improve the reproducibility of the work that we publish. This form provides structure for consistency and transparency in reporting. For further information on Nature Portfolio policies, see our Editorial Policies and the Editorial Policy Checklist.

Statistics

For all statistical analyses, confirm that the following items are present in the figure legend, table legend, main text, or Methods section.

n/a	Confirmed	
☐	☒	The exact sample size (*n*) for each experimental group/condition, given as a discrete number and unit of measurement
☐	☒	A statement on whether measurements were taken from distinct samples or whether the same sample was measured repeatedly
☐	☒	The statistical test(s) used AND whether they are one- or two-sided<br>*Only common tests should be described solely by name; describe more complex techniques in the Methods section.*
☒	☐	A description of all covariates tested
☐	☒	A description of any assumptions or corrections, such as tests of normality and adjustment for multiple comparisons
☐	☒	A full description of the statistical parameters including central tendency (e.g. means) or other basic estimates (e.g. regression coefficient) AND variation (e.g. standard deviation) or associated estimates of uncertainty (e.g. confidence intervals)
☐	☒	For null hypothesis testing, the test statistic (e.g. *F*, *t*, *r*) with confidence intervals, effect sizes, degrees of freedom and *P* value noted<br>*Give P values as exact values whenever suitable.*
☒	☐	For Bayesian analysis, information on the choice of priors and Markov chain Monte Carlo settings
☐	☒	For hierarchical and complex designs, identification of the appropriate level for tests and full reporting of outcomes
☐	☒	Estimates of effect sizes (e.g. Cohen's *d*, Pearson's *r*), indicating how they were calculated

Our web collection on statistics for biologists contains articles on many of the points above.

Software and code

Policy information about availability of computer code

Data collection	fastq-dll (v1.0.3)
Data analysis	Data analysis was performed using both commercial and open-source software. Commercial software included Trailmaker (https://app.trailmaker.parsebiosciences.com, Pipeline v1.4.0, Parse Biosciences; Seurat v5; SoupX v1.6.2; scds v1.18.0; cellRanger 1.1.0; SpacerRanger v2.1.1; Loupe Browser v8.1.2; OPTICS v1.3. Custom codes are available in: https://github.com/lnperez90/Perez-et-al-2025.

For manuscripts utilizing custom algorithms or software that are central to the research but not yet described in published literature, software must be made available to editors and reviewers. We strongly encourage code deposition in a community repository (e.g. GitHub). See the Nature Portfolio guidelines for submitting code & software for further information.

Data

Policy information about availability of data

All manuscripts must include a data availability statement. This statement should provide the following information, where applicable:

- Accession codes, unique identifiers, or web links for publicly available datasets
- A description of any restrictions on data availability
- For clinical datasets or third party data, please ensure that the statement adheres to our policy

Data availability
Raw sequencing data for single-nucleus RNA-seq (GSE306228) and spatial transcriptomics RNA-seq (GSE306418) generated in this study have been deposited in the Gene Expression Omnibus (GEO) and are available at https://www.ncbi.nlm.nih.gov/geo/query/acc.cgi?acc=GSE306228 and https://www.ncbi.nlm.nih.gov/geo/

query/acc.cgi?acc=GSE306418. Processed snRNA-seq data are available at the Broad Single Cell Portal https://singlecell.broadinstitute.org/single_cell/study/SCP3821 (SCP: SCP3821).

Code availability
All code used in this study is available at our GitHub repository (https://github.com/lnperez90/Perez-et-al-2025) and has been archived with Zenodo at https://doi.org/10.5281/zenodo.2279744127.

Research involving human participants, their data, or biological material

Policy information about studies with human participants or human data. See also policy information about sex, gender (identity/presentation), and sexual orientation and race, ethnicity and racism.

Reporting on sex and gender	NA. This study did not involve human participants or human data.
Reporting on race, ethnicity, or other socially relevant groupings	NA. This study did not involve human participants or human data.
Population characteristics	NA. This study did not involve human participants or human data.
Recruitment	NA. This study did not involve human participants or human data.
Ethics oversight	NA. This study did not involve human participants or human data.

Note that full information on the approval of the study protocol must also be provided in the manuscript.

Field-specific reporting

Please select the one below that is the best fit for your research. If you are not sure, read the appropriate sections before making your selection.

[x] Life sciences [ ] Behavioural & social sciences [ ] Ecological, evolutionary & environmental sciences

For a reference copy of the document with all sections, see nature.com/documents/nr-reporting-summary-flat.pdf

Life sciences study design

All studies must disclose on these points even when the disclosure is negative.

Sample size	No statistical method was used to predetermine sample size. Five independent biological replicas of adult Anableps retina were used for snRNA-seq, consistent with standard practice for snRNA-Seq dataset.
Data exclusions	Low-quality nuclei from the snRNA-seq dataset were excluded during preprocessing using SoupX, scds, and Seurat. We also performed two rounds of cluster exclusion to remove clusters representing immune contamination, smooth muscle, and residual doublet contamination, as described in the Methods section.
Replication	Five independent biological replicates were used for snRNA-seq of adult Anableps whole retina. Spatial transcriptomics was performed on single tissue section from Anableps eye (stage 5) and adult, and head (stage 3 and 4), and adult eye of Fundulus heteroclitus. HCR Gold RNA-FISH analysis were performed using at least two technical replicates for zebrafish (adult) and Anableps (adult and stage 3).
Randomization	For Anableps anableps samples used for eye developmental analysis, developmental stages were determined according to the description provided by Perez et al. (2017).
Blinding	Blinding was not applicable in this study. Experimental sample collection and molecular assays followed standardized protocols, and data analyses were conducted using automated bioinformatics pipelines, reducing the potential for bias.

Reporting for specific materials, systems and methods

We require information from authors about some types of materials, experimental systems and methods used in many studies. Here, indicate whether each material, system or method listed is relevant to your study. If you are not sure if a list item applies to your research, read the appropriate section before selecting a response.

Materials & experimental systems

n/a	Involved in the study	
☒	☐	Antibodies
☒	☐	Eukaryotic cell lines
☒	☐	Palaeontology and archaeology
☐	☒	Animals and other organisms
☒	☐	Clinical data
☒	☐	Dual use research of concern
☒	☐	Plants

Methods

n/a	Involved in the study	
☒	☐	ChIP-seq
☒	☐	Flow cytometry
☒	☐	MRI-based neuroimaging

Animals and other research organisms

Policy information about studies involving animals; ARRIVE guidelines recommended for reporting animal research, and Sex and Gender in Research

Laboratory animals	Anableps anableps, Fundulus heteroclitus, and Danio rerio were obtained from pet suppliers.
Wild animals	The study did not involve wild animals.
Reporting on sex	For snRNA-seq, adult Anableps anableps retinas were used. For spatial transcriptomics, we used adult eyes from Anableps anableps and Fundulus heteroclitus, as well as eyes or heads from eye developmental of Anableps at stages 3, 4, and 5. For HCR, we used eye sections from adult Anableps and zebrafish, and also stage 3 of Anableps.
Field-collected samples	The study did not involve field collections.
Ethics oversight	Animals were maintained and used in accordance with an approved Louisiana State University (LSU) Institutional Animal Care and Use committee (IACUC): Protocols IACUCAM-25-094 and IACUCAM-24-003.

Note that full information on the approval of the study protocol must also be provided in the manuscript.

Plants

Seed stocks	NA
Novel plant genotypes	NA
Authentication	NA
