# Supplementary material for: Evolutionary remodeling of the ventral retina enables aerial vision in the four-eyed fish Anableps anableps
Source: Nat Commun. 2026 Oct 14;17:11619. doi: 10.1038/s41467-026-78775-4 (PMC13754780; doi:10.1038/s41467-026-78775-4)
Supplement: Supplementary file 4 [file 41467_2026_78775_MOESM4_ESM.pdf]

Description of Additional Supplementary Files

Supplementary Data 1

Description: Spatial transcriptomics top 100 up-regulated genes among the clusters.

Supplementary Data 2

Description: OPTICS results.
